# Metal (Cu/Fe/Mn)-Doped Silicon/Graphite Composite as a Cost-Effective Anode for Li-Ion Batteries

**DOI:** 10.3390/nano12173004

**Published:** 2022-08-30

**Authors:** Arunakumari Nulu, Young Geun Hwang, Venugopal Nulu, Keun Yong Sohn

**Affiliations:** Department of Nanoscience and Engineering, Center for Nano Manufacturing, Inje University, 197 Inje-ro, Gimhae 50834, Gyeongnam-do, Korea

**Keywords:** silicon–graphite composites, transition metal doping, cost-effective anodes, Li-ion batteries, Li^+^ energy storage

## Abstract

Silicon is a worthy substitute anode material for lithium-ion batteries because it offers high theoretical capacity and low working potentials vs. Li^+^/Li. However, immense volume changes and the low intrinsic conductivity of Si hampers its practical applications. In this study, nano/micro silicon particles are achieved by ball milling silicon mesh powder as a scalable process. Subsequent metal (Cu/Fe/Mn) doping into nano/micro silicon by low-temperature annealing, followed by high-temperature annealing with graphite, gives a metal-doped silicon/graphite composite. The obtained composites were studied as anodes for Li-ion batteries, and they delivered high reversible capacities of more than 1000 mAh g^−1^ with improved Li^+^ diffusion properties. The full cells from these composite anodes vs. LiCoO_2_ cathodes delivered suitable energy densities for Li^+^ storage applications. The enhanced electrochemical properties are accredited to the synergistic effect of metal doping and graphite addition to silicon and exhibit potential for suitable Li^+^ energy storage applications.

## 1. Introduction

Lithium-ion batteries (LIBs) have many advantages over other energy storage devices, such as high energy density, low discharging rate, long cycling life, portability, and light weight, which makes them a promising technology for future energy storage applications [1,2,3,4]. The overall performance of LIBs is significantly influenced by the properties of electrode materials; hence, anode materials play a vital role in accomplishing high energy and power densities [5,6]. Commercial graphite is a conventional anode material for LIBs, owing to its high abundance, structural stability, low working potential, and long-term cyclability. However, its low theoretical capacity of 372 mAh g^−1^ is incapable of meeting current high energy demands, such as automotive applications, electric vehicles, energy storage devices, etc. [7,8]. Therefore, alternative anode materials with high theoretical capacities with low working potentials are necessary to meet the high energy and power density demands. Silicon (Si) is considered one of the promising anode materials owing to its high theoretical capacity of ~4200 mAh g^−1^ and low working potential of ~0.4 V vs. Li/Li^+^. Its environmentally friendly nature and high abundance further boost its practical applicability as an efficient anode material for LIBs [9,10,11]. However, massive volume expansion, low intrinsic conductivity, and an unstable solid electrolyte interphase (SEI) layer limit its practical applicability. Active Si undergoes severe structural and mechanical stress during Li^+^ alloying/dealloying reactions and leads to the loss of contact with the correct collector and causes a rapid decline in capacities [12,13].

To address these issues, several strategies have been employed to improve the Si-based anodes, including reducing the Si particle size from bulk to nanosize [14,15], and synthesizing different Si nanostructures to control the cracking and pulverization of Si [16,17]. Nanosize active materials benefit in alleviating structural breakdowns affected by the large strain. The large surface area provided by nanomaterials could afford a large reaction site for Li^+^ ions and thus retain superior specific capacities [18]. However, the large-scale material production of these nanostructures is highly impractical due to the requirement of sophisticated equipment and high synthesis expenditures. Preparation of nano/micro/sub-micron Si particles from bulk Si by high-energy ball milling is an affordable and cost-effective approach, which greatly reduces the production cost of Si [19,20]. Merging nanosized anode materials with carbon/carbonaceous materials greatly improves the electrochemical performance by relieving the volume changes and holding the electrical contact during the lithiation/delithiation process [18]. Introducing carbon materials as composites with Si and using protective carbon coatings are also effective ways to improve the performance of Si anodes [21,22,23]. The amount of carbon in the composite material plays a crucial role in stabilizing the Si anode performance by affording the required electrical conductivity and mechanical stability [24,25]. However, adding large amounts of carbon significantly reduces the overall specific capacity of the composite material by forming a dead layer on the surface of the electrode. Impurity doping such as boron (B) [26,27] and phosphorous (P) [28,29] is another contemporary way to overcome these disadvantages and advance the conductivity and ionic mobility of Si anodes. Ke et al. synthesized thin carbon shell and nitrogen/phosphorus co-doped two-dimensional (2D) carbon sheet dual encapsulate Si nanoparticles, which showed high reversible capacities of 592 mAh g^−1^ after 100 cycles at 200 mA g^−1^, indicating the excellent conductive support of nitrogen and phosphorous dopants along with carbon [30].

Recent reports reveal that transition metal alloying/doping/composites with Si improve its electrical conductivity and structural stability and are favorable in enhancing the overall electrochemical performance [31,32,33]. Hou et al. prepared ultrafine cobalt sulfide encapsulated multi-channel carbon nanofibers as anodes, which showed excellent electrochemical properties with 737 mAh g^−1^ capacities after 100 cycles at 200 mA g^−1^ [18]. Previous reports also suggested that the transition metal-doped Si nanoparticles showed improved electronic and magnetic properties [34,35]. Kim et al. prepared Al-doped Si film and studied it as anode material for LIBs. The Al-doped Si films showed better electrochemical properties than pristine Si [36]. Huang et al. prepared a NiSi_x_ intermetallic skin-coated Si anode, where NiSi_x_ acted as a protective layer, which exhibited improved cyclability with rate capabilities [37]. Lin et al. fabricated Cu-induced porous Si composite films as an anode, which showed excellent electrochemical properties [38]. Lee et al. prepared a graphite–FeSi alloy composite anode, where FeSi_2_ acted as a buffer matrix for active Si and exhibited good cyclability with high reversible capacities [39]. Bimodal nanoporous NiO@Ni-Si networks prepared by Wang et al. delivered excellent long-term cyclability with 1387 mAh g^−1^ capacities at 500 mA g^−1^ after 1000 cycles, where the Ni-Si network plays a crucial role in accommodating the large volume changes during cycling [40]. The composite of dual-shell Si/TiO_2_/CF prepared by Zeng et al. delivered 583 mAh g^−1^ specific capacity with 87.4% retention at 100 mA g^−1^ after 180 cycles, where the rigid TiO_2_ gives the required mechanical strength, and carbon affords excellent electrical conductivity to Si particles [41]. From our previous report, Co-doped Si nanoparticles also showed favorable electrochemical properties, along with improved Li^+^ diffusion properties [30]. Apart from Si anodes, other anode materials doped with Cu, Fe, and Mn such as Li_3_VO_4_, SnO_2_, TiO_2_, ZnFe_2_O_4_, etc., showed improved electrochemical properties compared to their pristine material [42,43,44,45,46]. All these results suggested that transition metal doping encourages Li^+^ kinetics, intrinsic conductivity, and structural stability, which favors the overall electrochemical properties of the host electrode.

In our previous work, the influence of metal doping on Si NPs was studied, where low doping concentrations of transition metals (Mn, Ni) were used to dope into Si nanoparticles [47]. However, the usage of Si NPs (commercially purchased) increases the production cost, which is a drawback for industrial applications. In this study, bulk Si is taken as precursor material instead of Si NPs, which is cost-effective, greatly reduces expenses, and encourages large-scale manufacture. The addition of graphite powder significantly enhances the stability of bulk Si in advancing a long and stable cycle life. Based on the above findings, considering a relatively cost-effective and scalable approach, we proposed a metal-doped Si/graphite composite as an anode material for LIBs. Here, three different transition metals (Cu, Fe, Mn) with low doping concentrations (0.5 at %) were used to dope into the Si host, and subsequently, construct a composite with graphite. The metal dopants in the composite enhanced the intrinsic conductivity of Si, as the graphite provides the required electrical conductivity and structural stability to Si. All prepared anodes delivered favorable electrochemical properties attributed to enriched electrical conductivity and structural stability provided by metal dopants and graphite in the composite material.

## 2. Experimental

### 2.1. Materials and Methods

Si powder (325 mesh, 99% trace metals basis, Sigma-Aldrich,Burlington, MA, USA), copper nitrate trihydrate (Cu(NO_3_)_2_·3 H_2_O—Sigma-Aldrich, Burlington, MA, USA), iron nitrate nonahydrate (Fe(NO_3_)_3_·9 H_2_O—Sigma-Aldrich, Burlington, MA, USA), manganese nitrate hexahydrate (Mn(NO_3_)_2_·6H_2_O—Sigma-Aldrich, Burlington, MA, USA), graphite powder (TIMCAL TIMREX^®^ SFG6, TIMCAL Ltd. Bodio, Leventina, Switzerland), and an ethanol solvent (≥99.5%, Sigma-Aldrich, Burlington, MA, USA) were purchased and used without further purification.

The metal-doped Si/graphite composite was prepared by a three-step process. Firstly, nano/micro Si particles were prepared from bulk Si (325 mesh). Later, the prepared nano/micro Si was doped with transition metals, followed by making a composite with graphite powder, yielding a transition metal-doped Si/graphite composite.

### 2.2. Preparation of Nano/Micro Si

To prepare nano/micro Si, 5 g of Si powder (325 mesh) was taken into a high-density polyethylene (HDPE) vial along with a few zirconium balls, with a ball-to-powder ratio of 50:1. Then, the vial was tightly closed with Teflon tape and placed in a ball mill. The milling was carried out for 48 h at the speed of 150 rpm at room temperature. The obtained Si powder with zirconium balls was sieved for about 2 h and the powder was collected, referred to as Si-48.

### 2.3. Fabrication of Transition Metal-Doped Nano/Micro Si

The prepared Si-48 powder was taken into a zirconium sample holder of a mini-mill and added with the desired amount (0.5 at%) of Cu precursor along with ~1 mL of ethanol. Then, the lid was placed and the milling was carried out for 1 h at a speed of 30 oscillations sec^−1^ to make a uniform slurry. The obtained slurry was taken into an alumina crucible and placed in a tubular furnace and heat-treated at 600 °C for 2 h at a heat rate of 3 °C min^−1^ under an argon atmosphere. When the furnace reached room temperature, the sample was collected and signed as SiCu by its metal dopant. Using this same approach, Fe-doped and Mn-doped Si materials were prepared and named SiFe and SiMn, respectively. All the prepared materials were used for further analysis.

### 2.4. Preparation of Metal-Doped Si/Graphite Composite

The preparation of metal-doped Si/graphite composite was as follows. Firstly, 425 mg of metal (Cu/Fe/Mn)-doped Si particles was taken into a mini-mill and 75 mg of graphite powder was added and milled for 30 min for thorough mixing of the two materials. Then, the mixture was transferred to an alumina crucible of a tubular furnace and heat-treated at 400 °C for 2 h at a heat rate of 5 °C min^−1^ in an argon atmosphere. After the furnace was cooled to room temperature, the samples were collected and named SiCuG, SiFeG, and SiMnG, referring to the presence of metal dopant and graphite. Si-48 was also added with graphite powder and prepared composite with the same experimental conditions to analyze the effect of metal dopants, and named SiG. All the prepared materials were used for further analysis.

### 2.5. Material Characterization

The crystal structure of the prepared materials was investigated by an X-ray diffractometer (XRD) using the Rigaku D/MAX-2200 (Rigaku corp., Tokyo, Japan) Ultima instrument equipped with Cu@Kα radiation (λ = 1.54056 Å) in the range of 10° < 2θ < 80° operating at 30 kV and 40 mA. The surface morphology and microstructures were analyzed by scanning electron microscopy SEM, JSM7000F (JEOL Ltd., Tokyo, Japan) and transmission electron microscopy TEM, JEM-2100F (JEOL Ltd., Tokyo, Japan). Raman spectroscopy was used to analyze the chemical composition of the samples. Thermogravimetric analysis (TGA) was operated from 25 °C to 800 °C to investigate the amount of carbon in the materials. X-ray photoelectron spectroscopy (XPS) on a PHI Quantear SXM (ULVAC-PHI Inc., Kanagawa, Japan) was employed to investigate the surface chemical states of the prepared materials. The Brunauer–Emmett–Teller (BET) method was applied to analyze the specific surface area of the prepared composite materials.

### 2.6. Electrochemical Measurements

The CR 2032 button-type coin cells were used to evaluate the electrochemical properties of all prepared materials. The working electrodes (SiCuG, SiFeG, and SiMnG) were prepared by adding 80% of active materials with 20% of polyamide-imide (PAI-(Typolymer. Co., Ltd. Torlon(R) Amideimide, 4000T-HV, Seoul, Korea)) in N-methyl pyrrolidone (NMP) as a binder. No conductive carbon additive was added due to the presence of graphite in the active material. For a comparison study, the other working electrodes (Si-48, SiCu, SiFe, and SiMn) were prepared by adding 68% active material with 12% super P as conductive carbon and 20% of PAI binder in NMP to match the amounts of Si, carbon, and binder. All components were taken into a mini-mill and milled for 15 min to make a uniform slurry. The resultant slurry was uniformly coated on a copper foil current collected with a 20 µm thickness by using the traditional doctor blade approach (wet film thickness: 0–3000 μm, controllable accuracy: 5 μm). Then, the electrodes were dried in the oven at 80 °C to evaporate the excess solvent, subsequently transferred to the vacuum oven, and dried at 200 °C for 3 h to activate the binder. After that, the electrodes were punched into 14 mm disks and used as working electrodes. The mass loading of the active material was ~2.6 mg.·cm^−1^, with an average thickness of 145 μm. All coin cells were fabricated in an Ar-filled glove box (H_2_O < 0.1 ppm and O_2_ < 1 ppm). Metallic lithium (MTI KOREA Corp., Seoul, Korea) with thickness: 0.17 mm, diameter: 16 mm was used as a counter electrode without prior surface cleaning. Then, 1 M LiPF6 in ethylene carbonate (EC), diethyl carbonate (DEC), and fluoroethylene carbonate (FEC) were used in a *v*/*v* ratio of 5:70:25. Polypropylene (PP Wellcos Corporation, Separator 2400) with a thickness of 27 μm and a diameter of 19 mm was used as the separator. The cyclability tests were carried out at 200 mA g^−1^, and the rate capability tests were conducted between 200 and 3200 mA g^−1^ applied currents, in the voltage range of 0.01 V–2.0 V. The redox behavior of the electrodes was investigated by employing cyclic voltammetry (CV) in the voltage window of 0.01 V–2.0 V at a scan rate of 0.1 mV s^−1^. Electrochemical impedance spectroscopy (EIS) was employed within the frequency range of 10 mHz to 10 kHz. For full cell preparation, 16 mm diameter disks of the prepared anodes were prelithiated by assembling half cells with Li metal as a counter electrode, with 1 M LiPF6 in EC:DEC:FEC (*v*/*v* ratio of 5:70:25) electrolyte, and a polypropylene separator. The cells were fully discharged to 0.01 V at 200 mA g^−1^. To prepare the cathode electrode, 90% commercial LiCoO_2_ (LCO, purity 99.5%, Thermo Fisher Scientific Inc., Waltham, MA, USA) powder was added to 5% Super-P carbon as a conductive additive and 5% PVDF in NMP as a binder. All components were thoroughly mixed using a mortar and pestle for 30 min to form a uniform slurry. The slurry was then coated on Al foil using the traditional doctor blade method (wet film thickness: 0–3000 microns, controllable accuracy: 5 μm) with a thickness of 45 μm and dried in an oven at 80 °C for 2 h. Later, the electrode was heat-treated in a vacuum oven at 100 °C for 1 h. Then, 14 mm diameter disks were punched and used as cathode electrodes. The specific capacity of the working electrode was calculated based on the weight of the active material.

## 3. Results and Discussion

### 3.1. Experimental Synthesis Mechanism

**Figure 1:**
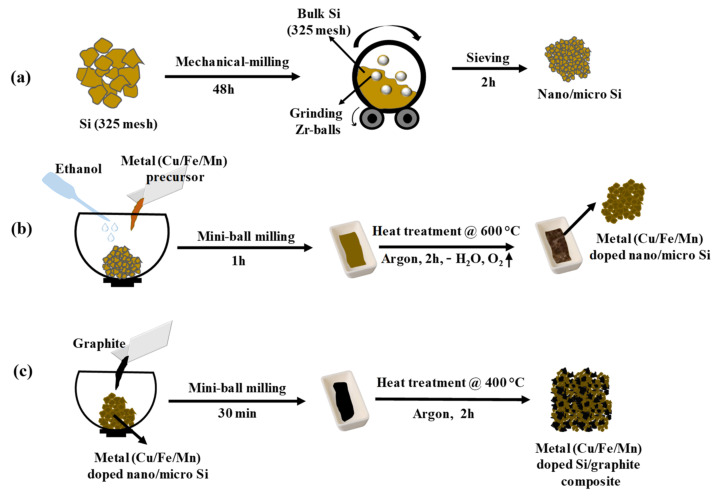
Schematic of step-by-step preparation of metal (Cu/Fe/Cu)-doped Si/graphite composite materials. (**a**) Preparation of nano/micro Si particles from bulk Si (325 mesh); (**b**) fabrication of metal doping into nano/micro Si particles; (**c**) metal (Cu/Fe/Mn)-doped Si/graphite composite preparation.

The schematic of the three-step preparation process of the metal (Cu/Fe/Mn)-doped Si/graphite composite is shown in Figure 1. The bulk Si (325 mesh) was taken into a ball mill with zirconium balls, and milling was carried out for 48 h, where bulk Si particles were crushed into nano/micro particles, as shown in Figure 1a. In the next step, the prepared nano/micro Si particles were thoroughly mixed with the desired amount of metal (Cu/Fe/Mn) nitrate precursor and ethanol solvent by using a mini-mill for 1 h. The obtained uniform slurry contains dissociated metal (Cu/Fe/Mn) and nitrate ions together with silicon particles and was transferred into an alumina crucible of a tubular furnace and subjected to thermal annealing at 600 °C for 2 h in an argon atmosphere. This process allows the successful metal (Cu/Fe/Mn) doping with minute nitrogen, while the excess amounts of solvent, water, and oxygen were excluded from the sample and giving metal (Cu/Fe/Mn)-doped nano/micro Si particles, as shown in Figure 1b. As shown in Figure 1c, metal (Cu/Fe/Mn)-doped nano/micro Si particles were mixed with graphite powder by using a mini-mill for 30 min to make a uniform mixture. Then, the mixture was taken into an alumina crucible and heat-treated at 400 °C for 2 h in argon. When the furnace was cooled to room temperature, the samples were collected and named SiCuG, SiFeG, and SiMnG, referring to their metal dopants.

### 3.2. Structure, Morphology and Component Analysis

**Figure 2:**
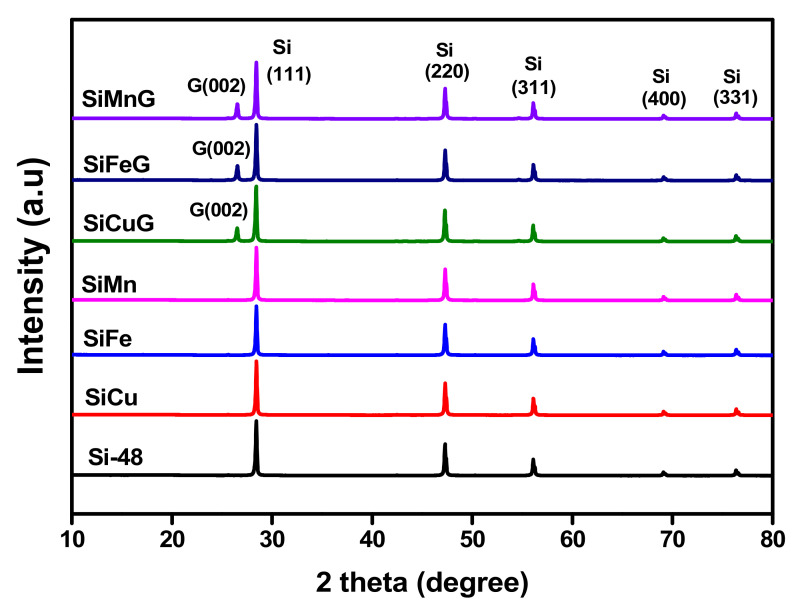
XRD patterns of Si-48, SiCu, SiFe, SiMn, SiCuG, SiFeG, and SiMnG materials.

**Figure 3:**
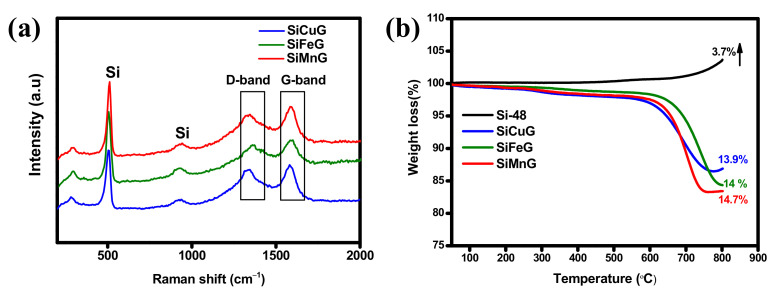
(**a**) Raman spectra of SiCuG, SiFeG, and SiMnG. (**b**) TGA results of Si-48, SiCuG, SiFeG, and SiMnG.

The XRD patterns of all prepared materials are shown in Figure 2. The sharp diffraction peaks at 2θ = 28.4°, 47.4°, 56.2°, 69.2°, and 76.3° correspond to the {111}, {220}, {311}, {400}, and {331} lattice planes of crystalline Si (JCPDS#27-1402), indicating the high crystallinity of the materials after the thermal annealing approach. The materials SiCuG, SiFeG, and SiMnG showed crystalline peaks around ~26.5°, corresponding to the {002} plane of graphite in the composite material. However, SiCuG, SiFeG, SiMnG, SiCu, SiFe, and SiMn materials did not show any peaks related to metal dopants, and this could be due to their low doping concentrations. No other impurity peaks were detected and are in agreement with the previous reports [10,21,30]. The elemental composition of the prepared composites was examined by Raman spectroscopy, and the obtained results for SiCuG, SiFeG, and SiMnG are shown in Figure 3a. The peaks around ~510 cm^−1^ and ~935 cm^−1^ are the characteristic peaks of crystalline Si and are observed for three materials [11,27]. The two broad peaks at 1345 and 1595 cm^−1^ in three materials correspond to the D-band and G-band, respectively. The D-band represents the structural defects and the G-band represents the graphitic carbon. These properties encourage electrical conductivity and structural stability and are beneficial for fast Li^+^ kinetics in the electrode materials [2,10,11]. The amounts of carbon present in the prepared composites were evaluated by using TGA. The TGA curves were measured from 50 °C to 800 °C at a heating rate of 10 °C min^−1^ in the air atmosphere. The resultant TGA curves are shown in Figure 3b. From the results, the weight of Si-48 is increased after 500 °C, demonstrating the partial oxidation on the surface of Si particles and the formation of the (SiO_x_) layer. The weight loss is observed for SiCuG, SiFeG, and SiMnG materials and could be accredited to the carbon oxidation in the air. The amounts of carbon in SiCuG, SiFeG, and SiMnG are 13.9%, 14%, and 14.7%, respectively, and are relatively close to the amount of carbon added in the preparation process, indicating the successful composite formation [25].

The microstructures of the prepared Si-48, SiCuG, SiFeG, and SiMnG were evaluated through SEM and TEM. The SEM images of Si-48 are shown in Figure S1a,b. From Figure S1a, we can observe the Si particles with different sizes ranging from ~150 nm to a few micrometers (µm), and the approximate particle sizes are shown in Figure S1b. The SEM images of SiCuG, SiFeG, and SiMnG are shown in Figure S1c, Figure S1e, and Figure S1f, respectively, where graphite flakes are thoroughly combined and incorporated with metal (Cu/Fe/Mn)-doped Si particles. The graphite flakes are shown with red arrows, whereas metal (Cu/Fe/Mn)-doped Si particles are shown with yellow arrows. A closer view of the images is shown in Figure S1d, Figure S1f, and Figure S1h, where graphite flakes in the composite are shown with white/black arrows, which are associated with metal-doped Si particles. These graphite flakes can give suitable conductivity and structural stability to Si particles and are favorable for delivering better electrochemical properties.

**Figure 4:**
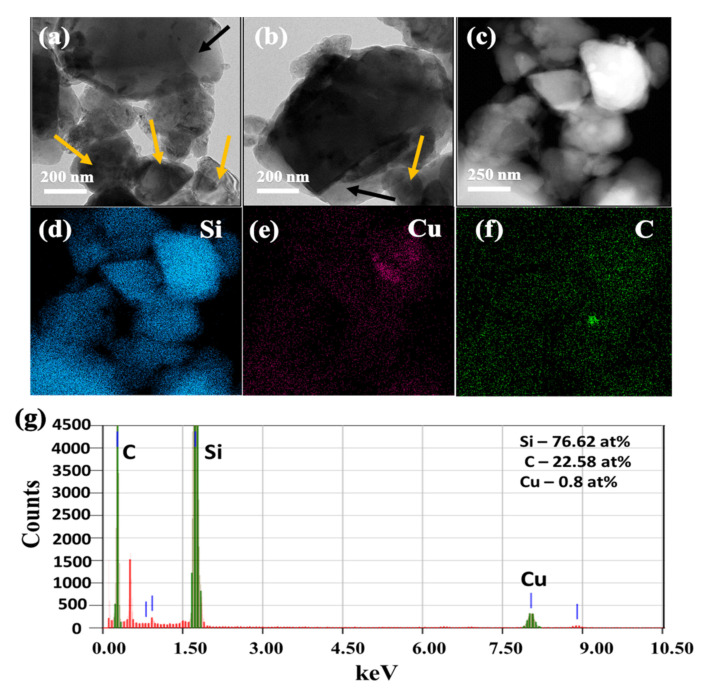
(**a**–**c**) TEM images, where yellow arrows represent Si particles and black arrows indicate graphite; (**d**–**f**) corresponding EDX elemental mapping of Si, Cu, and C of (**c**); (**g**) TEM-EDX spectra of SiCuG, where green colored lines indicate C, Si and Cu (from left to right), and red colored lines indicate Copper (Cu) from the TEM sample grid.

**Figure 5:**
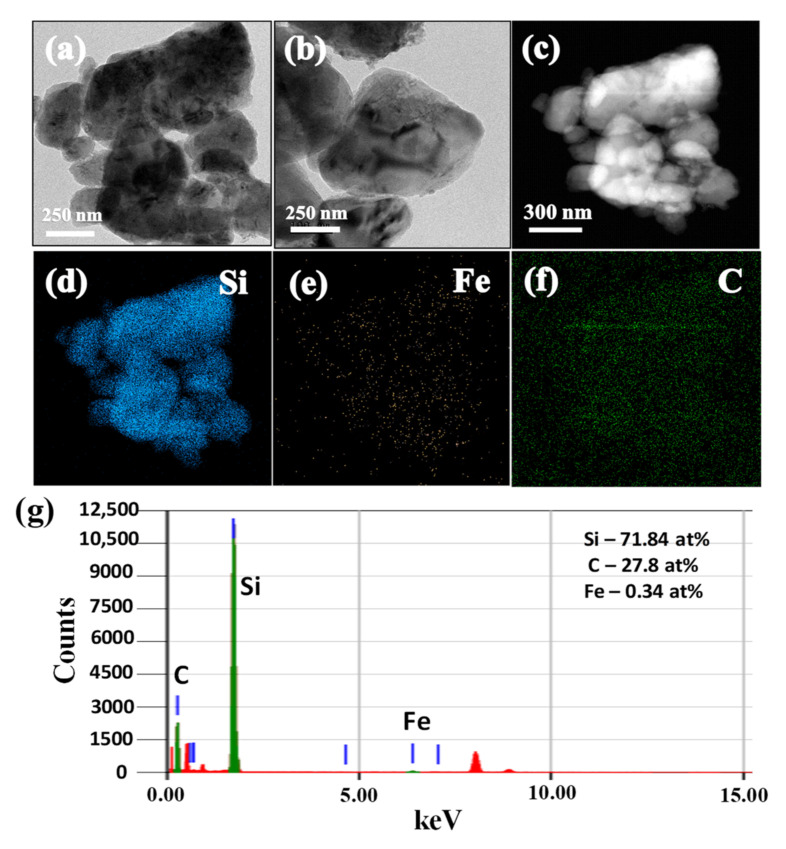
(**a**–**c**) TEM images; (**d**–**f**) corresponding EDX elemental mapping of Si, Fe, and C of (**c**); (**g**) TEM-EDX spectra of SiFeG, where green colored lines indicate C, Si and Fe (from left to right), and red colored lines indicate Copper (Cu) from the TEM sample grid.

**Figure 6:**
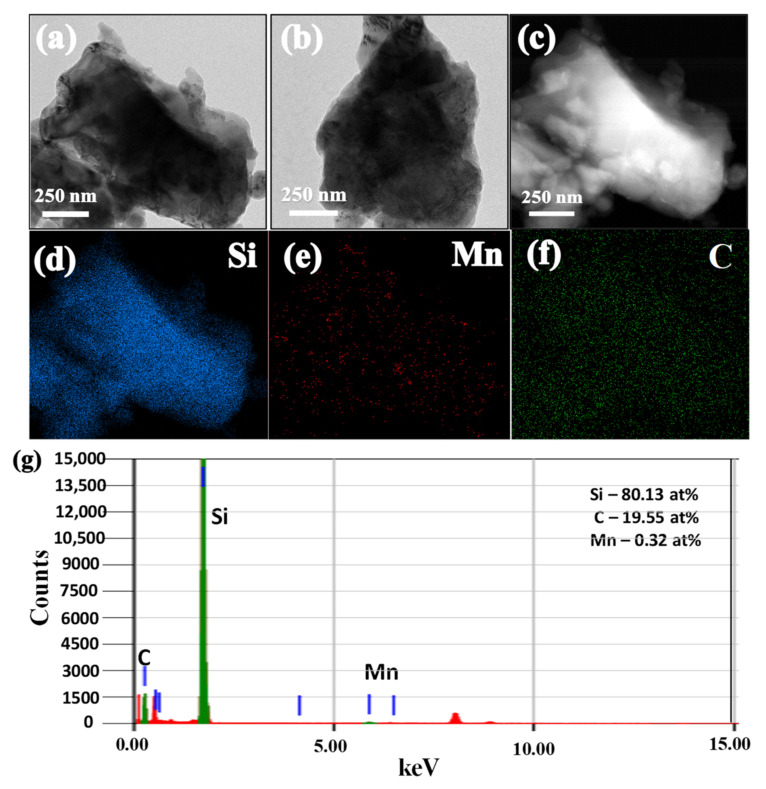
(**a**–**c**) TEM images; (**d–f**) corresponding EDX elemental mapping of Si, Mn, and C of (**c**); (**g**) TEM-EDX spectra of SiMnG, where green colored lines indicate C, Si and Mn (from left to right), and red colored lines indicate Copper (Cu) from the TEM sample grid.

The TEM images of SiCuG are shown in Figure 4a–c, where we can observe the agglomeration of nano/micro Si particles with graphite shown with yellow and black arrows, respectively. The TEM-EDX elemental mapping of Figure 4c is shown in Figure 4d–f with respect to the elements Si, Cu, and C, indicating their presence in the prepared material. The TEM-EDX spectra of SiCuG is shown in Figure 4g, indicating Si, C, and Cu with 76.62 at %, 22.58 at %, and 0.8 at %. The spectra show high amounts of Cu in the material compared to the precursor, and this could be due to the Cu grid used in TEM analysis. The TEM images of SiFeG are shown in Figure 5a–c, and those of SiMnG are shown in Figure 6a–c. The TEM-EDX elemental mapping of Figure 5c is shown in Figure 5d–f with respect to the elements Si, Fe, and C. Likewise, the TEM-EDX elemental mapping of Figure 6c is shown in Figure 6d–f with respect to the elements Si, Mn, and C. The TEM-EDX spectra of SiFeG and SiMnG are shown in Figure 5g and Figure 6g, respectively, showing their respective elements. The amounts of Fe and Mn are about 0.34 at % and 0.32 at % in SiFeG and SiMnG, respectively, which are relatively close to the doping concentrations. All these results significantly indicate the presence of metal dopants in their respective composite materials.

**Figure 7:**
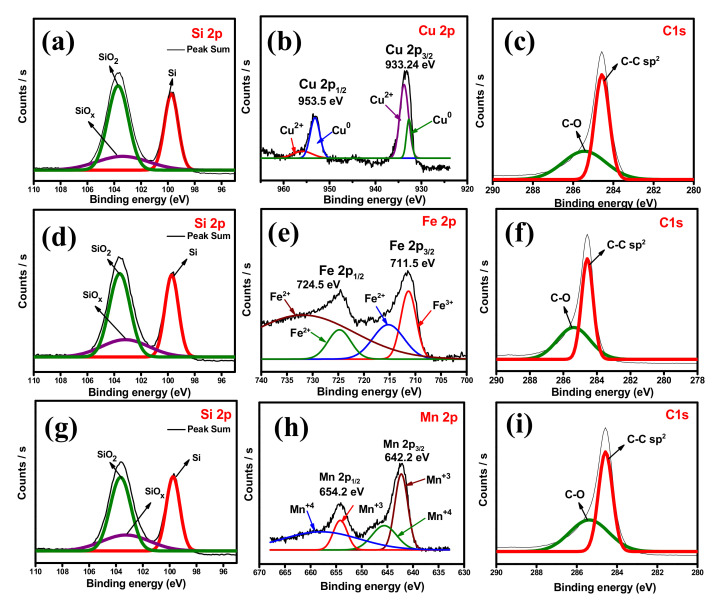
High-resolution XPS-spectra. (**a**) Si 2p, (**b**) Cu 2p, (**c**) C 1s of SiCuG. (**d**) Si 2p, (**e**) Fe 2p, (**f**) C 1s of SiFeG. (**g**) Si 2p, (**h**) Mn 2p, (**i**) C 1s of SiMnG.

The information about the surface chemical states of the prepared composites is determined by XPS, and the resultant spectrum is shown in Figure 7 and Figure S2. The survey spectrum of SiCuG is shown in Figure S2a, indicating that the composite is composed of Si, Cu, G, and O. The curve-fitting approach was used to distinguish the positions and chemical states of the elements. The high-resolution spectra of Si 2p are fitted to three peaks in Figure 7a. The peaks at binding energies around 99.7 and 103.7 eV are assigned to Si^0^ and Si^4+^, respectively. The broad peak between 99 and 104 eV can be ascribed to the commonly observed surface SiO_x_ layer of Si NPs [48]. Low-intensity Cu 2p peaks were observed due to the low doping concentration of Cu, and the high-resolution Cu 2p spectra are shown in Figure 7b and imply two main peaks located at 953.5 and 933.24 eV, attributed to Cu 2p_1/2_ and Cu 2p_3/2_, respectively. The peaks at binding energies 955.9 and 933.7 eV are attributed to Cu^2+^ peaks, and the binding energies at 953.1 and 932.7 eV correspond to Cu^0^ peaks and suggest the presence of Cu in the material [37]. The C 1s spectra shown in Figure 7c comprise two peaks at 284.5 and 285.4 eV related to the C-C bonds and C-O bonds, respectively [49]. The survey spectra of SiFeG and SiMnG are shown in Figure S2b and Figure S2c, respectively. Figure S2b shows binding energy peaks of Si, Fe, C, and O, and S2c shows binding energy peaks of Si, Mn, C, and O. The high-resolution Si 2p spectra of SiFeG and SiMnG are shown in Figure 7d and Figure 7g, respectively. In both Si 2p spectra, the peaks around ~99.7 and ~103.5 eV correspond to Si^0^ and Si^4+^, respectively, and the broad peak around 103 eV is ascribed to the SiO_x_ surface layer. The high-resolution Fe 2P spectra is shown in Figure 7e and shows two peaks at 724.5 and 711.5 eV binding energies, which reflect Fe 2p_1/2_ and Fe 2p_3/2_, respectively. The binding energies at 732.1, 724.6, and 715.3 eV are related to Fe^2+^ peaks, and the binding energy at 711.3 eV is attributed to Fe^3+^ and confirms the presence of Fe in SiFeG material [50]. The high-resolution Mn 2p spectra are shown in Figure 7h, where the peaks at 654.2 and 642.2 eV are assigned to Mn 2p_1/2_ and Mn 2p_3/2_, respectively. The peaks at binding energies at 658.2 and 645.6 eV are responsible for Mn^4+^ and the peaks at binding energies at 654.2 and 642.2 eV are related to Mn^3+^, implying the presence of Mn in SiMnG material [51]. The C 1s spectra of SiFeG and SiMnG materials are shown in Figure 7f and Figure 7i, respectively. Both spectra show binding energy peaks at around ~284.5 and 285.7 eV, implying C-C and C-O bonds, respectively [49]. The O 1s spectra of SiCuG, SiFeG, and SiMnG materials are shown in Figure S2d, Figure S2e, and Figure S2f, respectively. Three spectra show the O 1s peak at binding energy around ~533.2 eV, originating from the SiO_x_ surface layer [21]. The evaluated BET surface areas of the prepared Si-48, SiCuG, SiFeG, and SiMnG are 2.1, 6.1, 6.36, and 8.37 m^2^ g^−1^, respectively. For all the composite materials, the surface areas are slightly increased after the addition of graphite and are favorable for affording more active sites for Li^+^ ion reactions [21].

## 4. Electrochemical Evaluation in LIBs

**Figure 8:**
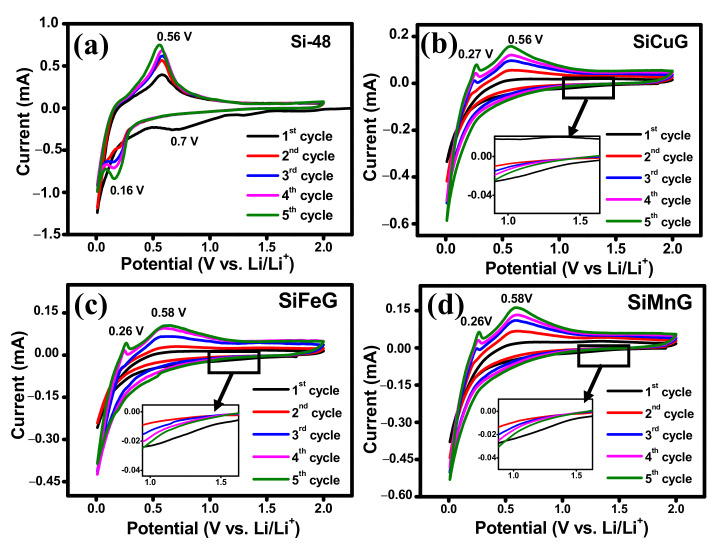
Cyclic voltammograms for 1–5 cycles of (**a**) Si-48, (**b**) SiCuG, (**c**) SiFeG, and (**d**) SiMnG vs. Li/Li^+^ as a counter electrode with 1 M LiPF_6_ in EC:DEC:FEC (*v*/*v* ratio of 5:70:25) at 0.1 mV s^−1^ scan rate in the potential window of 0.01–2.0 V.

Different electrochemical tests were carried out to investigate the electrochemical performance of the prepared electrode materials. To compare the performance of electrodes, all tests were conducted under the same conditions. The CV results for the first five cycles of Si-48, SiCuG, SiFeG, and SiMnG are shown in Figure 8a, 8b, 8c, and 8d, respectively, and SiCu, SiFe, and SiMn are shown in Figure S3a, Figure S3b, and Figure S3c, respectively. In the first lithiation of the cathodic scan, Si-48 shows a typical broad reduction peak around ~0.7 V, resulting from the SEI formation layer due to the electrolyte decomposition on the surface of the electrode material. the SiCuG, SiFeG, and SiMnG materials show a smaller extent of SEI formation, indicated by a small reduction peak around ~1.2 V (shown in the insets of Figure 8b–d). A possible reason could be because the formation of the SEI layer may be a sluggish process during the initial cycles, so the characteristic SEI reduction peaks were not obvious in the initial cycles of CV curves [52]. This SEI reduction peak disappeared in the following cycles, indicating stable SEI layer formation. The sharp peak at 0.05 V vs. Li^+^/Li in all electrodes corresponds to the alloying of Li^+^ in crystalline Si and the formation of an amorphous Li_x_Si phase. In the anodic scan, SiCuG, SiFeG, and SiMnG materials show two peaks at around ~0.26 V and ~0.58 V, which are related to the characteristic two-step dealloying process of Li_x_Si phase to metastable amorphous Si. However, in the anodic scan, Si-48 shows one sharp peak around 0.56 V, resulting from the dealloying of Li^+^ from the Li_x_Si phase and the formation of amorphous Si, indicating that dealloying in Si-48 is a slow and sluggish process. This occurrence is normally observed in many Si-based anodes. The increase in peak intensities in all electrodes increases with cycles due to the ongoing activation process of the active materials [25,45]. The CV results of SiCu, SiFe, and SiMn are similar to the CV curves of Si-48, indicating that the reaction path of the lithiation and delithiation process was not changed after metal doping into Si, due to inactivity of metal dopants towards Li-ions [30].

**Figure 9:**
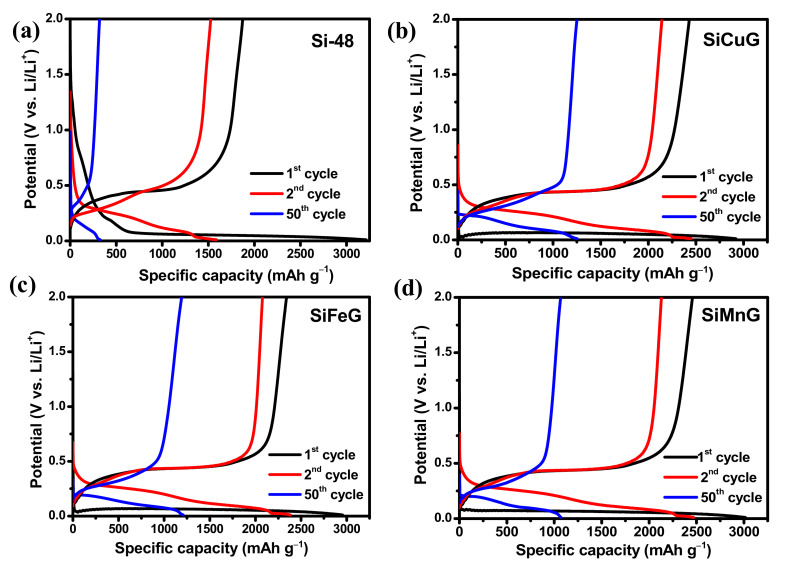
Voltage vs. specific capacity profiles for 1st, 2nd, and 50th cycles of (**a**) Si-48, (**b**) SiCuG, (**c**) SiFeG, and (**d**) SiMnG vs. Li/Li^+^ as counter electrode with 1 M LiPF_6_ in EC:DEC:FEC (*v*/*v* ratio of 5:70:25), at 200 mA g^−1^ in the potential window of 0.01–2.0 V.

The specific capacity vs. voltage profiles for the 1st, 2nd, and 50th cycles at a current density of 200 mA g^−1^ for Si-48, SiCuG, SiFeG, and SiMnG are shown in the Figure 9a, Figure 9b, Figure 9c, and Figure 9d, respectively. The long discharge plateau below 0.1 V in the first cycle in all electrodes is related to the alloying reaction of Li-ions with crystalline Si and the formation of the Li_x_Si phase. In the following cycles, the discharge plateaus are shifted to above ~0.22 V due to the formation of amorphous Si. The charge plateaus above ~0.5 V are in all electrodes, representing the dealloying reaction Li_x_Si phase to amorphous Si [12]. These charge/discharge profiles are in agreement with CV profiles. The first discharge/charge capacities of Si-48, SiCuG, SiFeG, and SiMnG are 3219/1874, 2919/2430, 2954/2340, and 2915/2456 mAh g^–1^ with Coulombic efficiencies (CEs) of 58.2%, 83.2%, 79.2%, and 84.2%, respectively. The irreversible capacity loss is accompanied by the formed SEI layer on the electrode’s surface. The lower CE% of Si-48 could be due to the formation of a thick SEI layer, and is consistent with CV results (Figure 8). On the other hand, the prepared anode materials showed better initial CE because of the thin SEI layer formation resulting from the metal dopant, which controls the Si volume expansion, and the graphite carbon, which maintains the overall structural stability. Furthermore, Si-48 capacities declined rapidly and reached 321/312 mAh g^−1^ with 97% CE after 50 cycles, but the prepared SiCuG, SiFeG, and SiMnG electrodes delivered 1253/1244, 1208/1197, and 1170/1165 mAh g^−1^ specific capacity with 99.2%, 99%, and 99.5% CE, respectively. The discharge/charge profiles for the 1st, 2nd, and 50th cycles for SiCu, SiFe, and SiMn are shown in Figure S4a, Figure S4b, and Figure S4c, respectively. These materials also show similar capacity vs. voltage profiles, indicating the same reaction path as other prepared materials.

**Figure 10:**
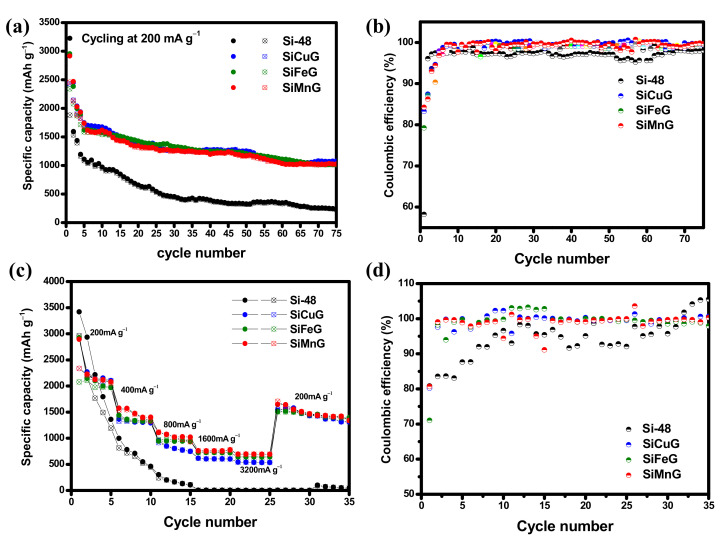
(**a**,**b**) Cyclability and respective Coulombic efficiency at 200 mA g^−1^ in the potential window of 0.01–2.0 V. (**c**,**d**) Rate capability and respective Coulombic efficiency results at applied currents of 200–3200 mA g^−1^ in the potential window of 0.01–2.0 V of Si-48, SiCuG, SiFeG, and SiMnG vs. Li/Li^+^ as counter electrode with 1 M LiPF_6_ in EC:DEC:FEC (*v*/*v* ratio of 5:70:25) electrolyte.

The long-term cyclic stability and CEs were evaluated for Si-48, SiG, SiCuG, SiFeG, SiMnG, SiCu, SiFe, and SiMn materials at applied currents of 200 mA g^−1^ within the voltage of 0.01–2.0 V. The cyclability and CE results for Si-48, SiCuG, SiFeG, and SiMnG are shown in Figure 10a,b, and those for SiG, SiCu, SiFe, and SiMn are shown in Figure S5a,b. From Figure 10a, Si-48 shows a higher initial capacity compared with the other three electrodes, but these capacities declined rapidly with the number of cycles. After 75 cycles, Si-48 reached 226/222 mAh g^−1^ with 98.2% CE and 23.4% capacity retention compared with the 10th cycle. The less stability and poor capacities could be due to the massive volume changes during lithium reactions, leading to structural destruction and peeling of active materials from the copper current collector. On the other hand, SiCuG, SiFeG, and SiMnG material showed better cyclic stabilities compared to Si-48. After 75 cycles, SiCuG, SiFeG, and SiMnG delivered 1075/1072, 1015/1009, and 1025/1022 mAh g^−1^ capacity with 99.7%, 99.4%, and 99.7% CE with 64.2%, 64.4%, and 63.3% capacity retention (compared with 10th cycle), respectively. From Figure S5a, Si-48, SiCu, SiFe, and SiMn materials show similar cyclability results. However, compared with Si-48, the prepared SiCu, SiFe, and SiMn materials show slight stable cyclability. After 75 cycles, SiCu, SiFe, and SiMn materials delivered 355/342, 417/412, and 437/431 mAh g^−1^ capacity with 61.3%, 67.6%, and 52.5% capacity retention (compared with the 10th cycle), respectively. From the cycling results of all prepared materials, it is evident that SiCu, SiFe, and SiMn show better cyclability results, and this could be due to the enhanced intrinsic conductivity and structural stability given by the metal dopants, which are helpful for faster Li^+^ reactions. The SiG material also showed better cyclability compared to Si-48, which could be due to the adequate conductivity and structural stability provided by the graphite. However, after 10 cycles, SiG also showed declined capacities. After 30 cycles, the SiG electrode managed to deliver 935/916 mAh g^−1^ specific capacities, which is less compared to SiCuG, SiFeG, and SiMnG electrodes. This result suggests that only the addition of graphite into Si-48 is not sufficient to attain stable capacities. On the other hand, SiCuG, SiFeG, and SiMnG materials showed much better cyclability results compared with SiCu, SiFe, and SiMn materials. These results suggest that metal doping into nano/micro Si is insufficient to afford the required structural stability and conductivity to nano/micro Si particles and the need for graphite carbon. The graphite in the composite successfully afforded the required conductivity and structural and mechanical stability to nano/micro Si and encouraged stable SEI formation on the electrode’s surface during harsh Li^+^ reactions. The synergetic properties of metal dopants and graphite together promote faster Li^+^ diffusion into the active materials and encourage enhanced electrochemical properties. The rate capability results were evaluated for Si-48, SiCuG, SiFeG, and SiMnG electrodes at wide applied current densities from 200 to 3200 mA g^−1^ in the voltage window of 0.01 V to 2.0 V, and the resultant specific capacity vs. cycle number plots and CEs are depicted in Figure 10c and Figure 10d, respectively. For all prepared materials, the specific capacities are decreased with increasing applied current densities. All prepared SiCuG, SiFeG, and SiMnG showed stable rate capability results compared to Si-48, which failed at higher current densities. Si-48 delivered 3418, 996, and 301 mAh g^−1^ at applied currents of 200, 400, and 800 mA g^−1^, respectively. After that, the rate capacities dropped quickly and reached single-digit capacities at 1600 mA g^−1^ and failed to sustain at higher current densities. On the contrary, the prepared anodes SiCuG/SiFeG/SiMnG delivered 2910/2924/2893, 1366/1440/1575, 955/943/1106, 615/729/761, and 543/642/695 mAh g^−1^ capacities at 200, 400, 800, 1600, and 3200 mA g^−1^ applied currents, respectively. The rate capability results suggest that the metal dopants and graphite in the composite material play a critical role in enhancing the conductivity and affording the structural flexibility of nano/micro Si particles. The specific capacities of all prepared anode materials at different cycles are summarized in Table 1.

**Figure 11:**
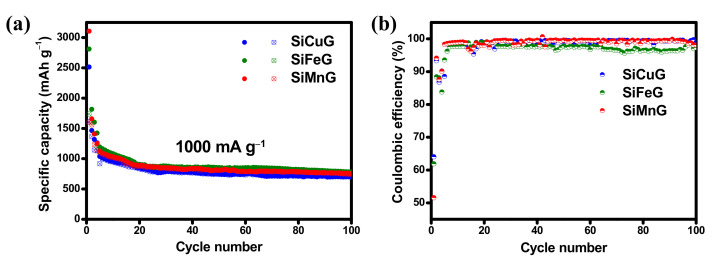
(**a**,**b**) Cyclability and respective Coulombic efficiency results of SiCuG, SiFeG, and SiMnG vs. Li/Li^+^ as counter electrode with 1 M LiPF_6_ in EC:DEC:FEC (*v*/*v* ratio of 5:70:25) electrolyte at 1000 mA g^−1^ in the potential window of 0.01–2.0 V.

The long cyclability and CEs of prepared materials were evaluated by cycling at high currents of 1000 mA g^−1^, and the results are shown in Figure 11a and Figure 11b, respectively. The three electrodes showed declined capacities in the initial cycles, but after 20 cycles, the capacities are stabilized. In the initial cycle, SiCuG, SiFeG, and SiMnG delivered 2514/1609, 2810/1740, and 3105/1600 mAh g^−1^ capacities with 64%, 61%, and 51% CE. After 100 cycles, the materials managed to deliver 704/696, 779/758, and 754/740 mAh g^−1^ specific capacities with 98.8%, 97.3%, and 98.1% CE, respectively. The capacity retention values compared with the 10th cycles are 73.6%, 72%, and 72.4%, respectively, and suggest the highly stable microstructures of metal-doped Si/G composite even at higher applied currents.

**Figure 12:**
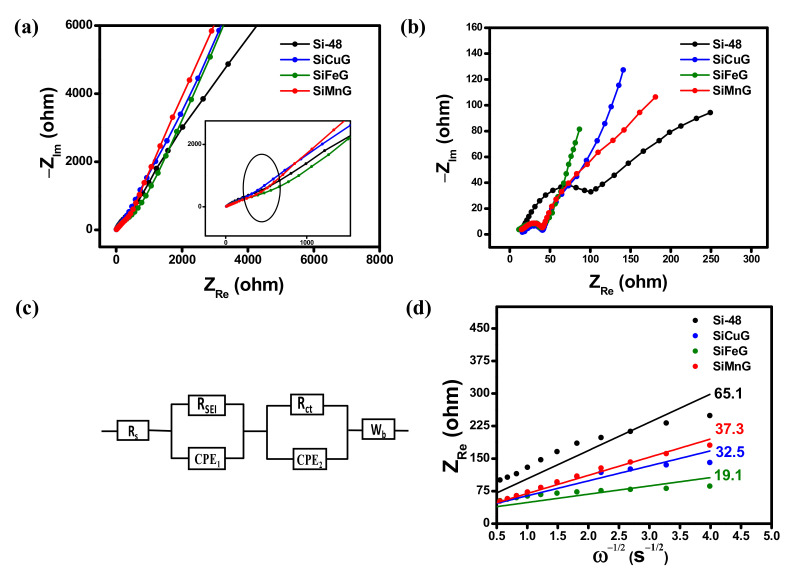
Electrochemical impedance spectroscopy results (**a**) before cycling and (**b**) after 25 cycles at 500 mA g^−1^. (**c**) Equivalent circuit; (**d**) Z_Re_ vs. ω^−1/2^ plots of Si-48, SiCuG, SiFeG, and SiMnG vs. Li/Li^+^ as counter electrode with 1 M LiPF_6_ in EC:DEC:FEC (*v*/*v* ratio of 5:70:25) electrolyte.

The Li^+^ kinetics between electrode and electrolyte were analyzed by employing EIS. The resultant Nyquist plots for Si-48, SiCuG, SiFeG, and SiMnG electrodes before and after 25 charge/discharge cycles at 500 mA g^−1^ are displayed in Figure 12a and Figure 12b, respectively. All three electrodes exhibited a depressed semicircle in the mid- and high-frequency regions related to the charge transfer resistance between the electrode and the electrolyte interface. The inclined line in the low-frequency region corresponds to the lithium diffusion process [53]. The equivalent circuit model in Figure 12c consists of solution resistance (R_s_), SEI layer resistance (R_SEI_), charge transfer resistance (R_ct_), constant phase elements (CPE_1_, CPE_2_), and Warburg impedance (W_b_). From Figure 12a, all electrodes exhibited R_ct_ of more than 500 Ω (shown in the inset Figure 10a). After 25 charge/discharge cycles at 500 mA g^−1^, the R_ct_ values are greatly decreased due to the wetting process of electrolyte into the electrodes and electrode kinetics with Li-ions. From Figure 12b, Si-48 showed 106.5 Ω, which is quite high compared to the other three prepared electrodes, as SiCuG, SiFeG, and SiMnG showed much lower R_ct_ values of 40.3, 38.2, and 40.7 Ω, respectively, and could be attributed to faster Li^+^ diffusion and enhanced electrical conductivity afforded by the metal dopants and graphite in the composite materials. The values of R_s_, R_SEI_, and R_ct_ values after 25 cycles are summarized in Table 2. The evaluation of Li^+^ diffusion properties, Z_Re_ vs. ω^−1/2^, is plotted and depicted in Figure 12d. From the plot, Z_Re_ linearly depends on ω^−1/2^ in the low-frequency region. The following equations were used to calculate the diffusion coefficient (D) of Li^+^ into the electrode materials.

Z_Re_ = R_s_ + R_SEI_ + R_ct_ + σ_w_ ω^−1/2^ (1)

D = R^2^ T^2^/2 A^2^ F ^4^ C^2^ σ_w_^2^ (2)

Here, R is the gas constant, T is the absolute temperature, C is the molar concentration of Li^+^ ions, A is the electrode area, and F is the Faraday constant. The slope of Z_Re_ vs. ω^−1/2^ plots gives the Warburg impedance coefficients σ_w._ The lower slope values yield higher diffusion coefficients and suggest better diffusion properties. The calculated Warburg factors for Si-48, SiCuG, SiFeG, and SiMnG are 65.1, 32.5, 19.1, and 37.3 Ω s^−1/2^, respectively. The calculated D values from Equation (2) for each electrode material are summarized in Table 2. All prepared materials show higher D values compared to Si-48, demonstrating the faster Li^+^ kinetics owing to enhanced conductivity, more ionic channels due to metal dopants, structural stability, and flexibility afforded by the graphite.

To investigate the structure and morphology changes of the Si-48, SiCuG, SiFeG, and SiMnG materials, the cells were carefully disassembled after 20 charge/discharge cycles, and the anode electrodes were collected. To remove the SEI layer, the electrodes were washed with anhydrous dimethyl carbonate solution and dried in an oven. Later, the electrode materials were carefully collected for SEM analysis. Figure S6 shows the SEM images of Si-48 and Figures S7–S9 show the SEM images, SEM-EDX elemental mapping, and spectra of SiCuG, SiFeG, and SiMnG, respectively. From Figure S6, the Si-48 particles are aggregated to a larger extent and no individual particles have appeared (as shown in Figure S1b), which results in structural damage and leads to declined specific capacities with cycle number (showed in Figure 10a). From the SEM images of SiCuG, SiFeG, and SiMnG, Si particles and graphite are agglomerated to a small extent. The SEM-EDX mapping and EDX spectra results also specify the presence of Si, metal (Cu/Fe/Mn) dopants, and C in their respective materials. These results suggest the favorable structural and mechanical stability of metal-doped Si/graphite composite materials even after 20 charge/discharge cycles.

**Figure 13:**
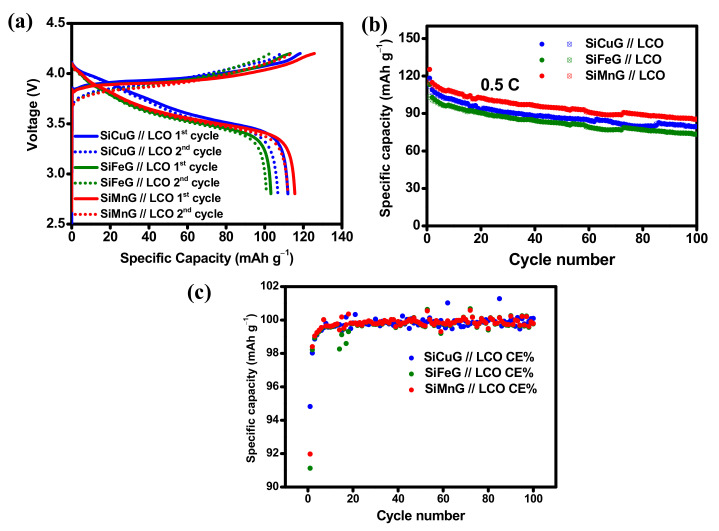
(**a**) Potential vs. specific capacity plots for 1st and 2nd cycles. (**b**) Cyclability, (**c**) Coulombic efficiency results of SiCuG // LCO, SiFeG // LCO, and SiMnG // LCO full cells with 1 M LiPF_6_ in EC:DEC:FEC (*v*/*v* ratio of 5:70:25) electrolyte at 0.5 C, in the potential window of 2.8–4.2 V, respectively.

The practical applicability of the prepared anodes SiCuG, SiFeG, and SiMnG are evaluated by fabricating full cells. The full cells were assembled with a commercial LiCoO_2_ (LCO) cathode. The fundamental electrochemical properties such as voltage profiles, cyclability, and rate capability results of the LCO cathode are shown in Figure S10a, Figure S10b, and Figure S10c, respectively. The cyclability tests were carried out at 0.5 C (1 C = 140 mA g^−1^, based on the cathode active material), and the rate capability test was carried out from 0.1 C to 5 C. After 100 cycles, the LCO cathode delivered 110.6/109.1 mAh g^−1^ capacity with 98.6% CE, and at the high rate of 5 C, the LCO cathode delivered 51.8/48.9 mAh g^−1^ capacity, indicating suitable cyclic stability and rate capability, suggesting LCO as a strong cathode material for LIBs. For full cell fabrication, the N/P ratio was fixed at 1.102, 1.142, and 1.083 for SiCuG, SiFeG, and SiMnG materials, respectively, to match the capacities of cathode and anode active materials. Before fabricating full cells, the prepared anodes (SiCuG, SiFeG, and SiMnG) were prelithiated up to two complete cycles, followed by complete discharge to 0.01 V at a current density of 200 mA g^−1^. After prelithiation, the cells were disassembled carefully in a glove box, and prelithiated anodes were used in full cell fabrication with the LCO cathode. The cyclability tests for full cells were carried out at 0.5 C (1 C =140 mA g^−1^, based on the cathode active material), and the results are shown in Figure 13. The 1st and 2nd voltage vs. specific capacity plots, cyclability, and CE results of SiCuG, SiFeG, and SiMnG are depicted in Figure 13a, Figure 13b, and Figure 13c, respectively. After 100 cycles, the full cells delivered 79.5, 73.5, and 85.5 mAh g^−1^ capacities with more than 99.5% CE with 79.5%, 72%, and 75% of capacity retention (vs. 2nd cycle charge capacity), respectively. Even though the electrode SiFeG shows a better diffusion coefficient (Table 2), it shows little lesser capacities compared to SiCuG and SiMnG. This could be due to the N/P ratio being a little higher than the other two electrodes, which significantly affects the full cell performance due to the availability of lower Li^+^ ions compared to the other two electrodes. Detailed observations revealed that three prepared composites show little differences in half cell and full cell performances. These small differences could be attributed to the individual metal dopants’ physical and chemical properties such as electrical conductivity, resistivity, how effectively they doped, the internal bonding of the metal matrix with host Si and/or with surface oxygen, etc. These factors could cause differences in the overall performance of the composite material [54]. The calculated energy densities for SiCuG // LCO, SiFeG // LCO, and SiMnG // LCO are 302, 297.5, and 314 Wh.·kg^−1^, respectively, with an average voltage of 3.5 V. The energy densities are reasonable for materials prepared from bulk Si particles by a low-cost and affordable approach and are most suitable and acceptable for Li-ion energy storage applications.

## 5. Conclusions

In this study, we successfully prepared a transition metal (Cu, Fe, Mn)-doped nano/micro silicon/graphite composite by employing a cost-effective and scalable approach. In the prepared composite, the doped metals endure significant volume changes by forming an inactive phase and enhancing the intrinsic conductivity. On the other hand, graphite acted as a tough framework and afforded structural stability along with improved conductivity to the nano/micro silicon particles. All these key factors favor the composite electrodes in achieving more than 1100 mAh g^−1^ capacity after 75 cycles at 200 mA g^−1^, and more than 700 mAh g^−1^ after 100 cycles at 1000 mA g^−1^. The full cells prepared from prelithiated anodes (SiCuG, SiFeG, and SiMnG) and the LiCoO_2_ cathode delivered 302, 297.5, and 314 Wh.·kg^−1^ energy densities, respectively, with an average voltage of 3.5 V. We believe that the successful fabrication of prepared materials by a simple and cost-effective approach benefits the insight into the development of next-generation, low-cost advanced battery materials and signifies a promising path for practical applicability.

## Figures and Tables

**Table 1:** Summarized specific capacities of all prepared anode materials at different cycles.

Cells	1st CyclećmAh g^−1^	2nd CyclećmAh g^−1^	30th CyclećmAh g^−1^	75th CyclećmAh g^−1^
Si-48 vs. Li^+^/Li	3219/1874	1588/1526	449/437	226/222
SiG vs. Li^+^/Li	2732/2207	1905/1845	935/916	-----------
SiCu vs. Li^+^/Li	3154/2490	1783/1446	442/440	355/342
SiFe vs. Li^+^/Li	3096/2527	1841/1642	413/411	417/412
SiMn vs. Li^+^/Li	3172/2311	1812/1503	474/471	437/431
SiCuG vs. Li^+^/Li	2919/2430	2450/2143	1268/1261	1075/1072
SiFeG vs. Li^+^/Li	2954/2340	2382/2077	1330/1319	1015/1009
SiMnG vs. Li^+^/Li	2915/2456	2469/2129	1257/1252	1025/1022

**Table 2:** Summarized impedance values (after cycling) and calculated diffusion coefficients.

Sample	R_s_ (Ω)	R_SEI_ (Ω)	R_ct_ (Ω)	D (cm^2^ s^−1^)
Si-48	5.6	22.3	106.5	3.53 × 10^−12^
SiCuG	11.3	15.2	40.3	1.41 × 10^−11^
SiFeG	8.4	14.8	38.2	4.09 × 10^−11^
SiMnG	12.6	15.3	40.7	1.07 × 10^−11^

## Data Availability

Not applicable.

## Supplementary Materials

The following supporting information can be downloaded at: https://www.mdpi.com/article/10.3390/nano12173004/s1, Figure S1: SEM images (a) & (b) Si- 48, (c) & (d) SiCuG, (e) & (f) SiFeG, and (g) & (h) SiMnG respectively; Figure S2: XPS-Spectra (a), (b), (c) survey scan spectra, (d), (e), (f) O 1S spectra of SiCuG, SiFeG, and SiMnG respectively; Figure S3: Cyclic voltammograms of (a) SiCu (b) SiFe and (c) SiMn vs. Li/Li^+^ counter electrode with 1M LiPF_6_ in EC: DEC: FEC (*v*/*v* ratio of 5:70:25) electrolyte at 0.1 mV s^-1^ scan rate, in the potential window of 0.01–2.0 V; Figure S4 : Voltage vs. specific capacity plots for 1^st^, 2^nd^, and 50^th^ cycles (a) SiCu (b) SiFe and (c) SiMn vs. Li/Li^+^ as a counter electrode with 1M LiPF6 in EC: DEC: FEC (*v*/*v* ratio of 5:70:25), at 200 mA g^-1^ in the potential window of 0.01–2.0 V; Figure S5: (a) cyclability (b) coulombic efficiency results of Si-48, SiCu, SiFe, SiMn and SiG vs. Li/Li^+^ as counter electrode with 1M LiPF_6_ in EC: DEC: FEC (*v*/*v* ratio of 5:70:25) electrolyte at 200 mA g^-1^ in the potential window of 0.01–2.0 V; Figure S6: SEM images of Si-48 after 20 cycles at different magnifications; Figure S7: (a) and (b) SEM images (c) SEM-EDX elemental mapping of image (b) with respective Si, C, and Cu elements overlap (d) Si mapping (e) C mapping (f) Cu mapping (g) SEM-EDS spe ctra of image (b); Figure S8: (a) and (b) SEM images (c) SEM-EDX elemental mapping of image (b) with respective Si, C, and Fe elements overlap (d) Si mapping (e) C mapping (f) Fe mapping (g) SEM-EDS spectra of image (b); Figure S9: (a) and (b) SEM images (c) SEM-EDX elemental mapping of image (b) with respective Si, C, and Mn elements overlap (d) Si mapping (e) C mapping (f) Mn mapping (g) SEM-EDS spectra of image (b); Figure S10: (a) Potential vs. specific capacity plots (b) cyclability (c) rate capability results of LCO vs. Li/L^+^ as a counter electrode at 0.5 C, in the voltage window of 3.0–4.2 V.

## References

[1] Tarascon J.M., Armand M. (2001). Issues and challenges facing rechargeable lithium batteries. Nature.

[2] Li M., Hou X.H., Sha Y.J., Wang J., Hu S.J., Liu X., Shao Z.P. (2014). Facile spray-drying/ pyrolysis synthesis of core-shell structure graphite/silicon-porous carbon composite as a superior anode for Li-ion batteries. J. Power Sources.

[3] Zubia G., López R.D., Carvalho M., Pasaoglu G. (2018). The lithium-ion battery: State of the art and future perspectives. Renew. Sustain. Energy Rev..

[4] Deng D. (2015). Li-ion batteries: Basics, progress, and challenges. Energy Sci. Eng..

[5] Heidari E.K., Gol A.K., Sohi M.H., Ataie A. (2018). Electrode materials for lithium-ion batteries: A review. J. Ultrafine Grained Nanostruct. Mater..

[6] An Y.L., Fei H.F., Zeng G.F., Ci L.J., Xiong S.L., Feng J.K., Qian Y.T. (2018). Green, scalable, and controllable fabrication of nanoporous silicon from commercial alloy precursors for high-energy lithium-ion batteries. ACS Nano.

[7] Li H., Huang X., Chen L., Wu Z., Liang Y. (1999). A high capacity nano-Si composite anode material for lithium rechargeable batteries. Electrochem. Solid-State Lett..

[8] Sun Y.K., Myung S.T., Park B.C., Prakash J., Belharouak I., Amine K. (2009). High-energy cathode material for long-life and safe lithium batteries. Nat. Mater..

[9] Baggetto L., Niessen R.A.H., Notten P.H.L. (2009). On the activation and charge transfer kinetics of evaporated silicon electrode/electrolyte interfaces. Electrochim. Acta.

[10] Li X., Wu M., Feng T., Xu Z., Qin J., Chen C., Tu C., Wang D. (2017). Graphene enhanced silicon/carbon composite as anode for high-performance lithium-ion batteries. RSC Adv..

[11] Nulu A., Nulu V., Sohn K.Y. (2020). Comparative study of different silicon/carbon nanocomposites as anode electrodes for li-ion batteries. Sci. Adv. Mater..

[12] Yang J., Wang B.F., Wang K., Liu Y., Xie J.Y., Wen Z.S. (2003). Si/C composites for high capacity lithium storage materials. Electrochem. Solid-State Lett..

[13] Kasavajjula U., Wang C., Appleby A.J. (2007). Nano- and bulk-silicon-based insertion anodes for lithium-ion secondary cells. J. Power Sources.

[14] Nulu A., Nulu V., Sohn K.Y. (2020). Silicon and porous MWCNT composite as high capacity anode for lithium-ion batteries. Korean J. Chem. Eng..

[15] Liu X.H., Zhong L., Huang S., Mao S.X., Zhu T., Huang J.Y. (2012). Size-dependent fracture of silicon nanoparticles during lithiation. ACS Nano.

[16] Chen Q., Zhu R., Liu S., Wu D., Fu H., Zhu J., He H. (2018). Self-templating synthesis of silicon nanorods from natural sepiolite for high-performance lithium-ion battery anodes. J. Mater. Chem. A.

[17] Park M.H., Kim M.G., Joo J., Kim K., Kim J., Ahn S., Cui Y., Cho J. (2009). Silicon nanotube battery anodes. Nano Lett..

[18] Hou Z., Jiang M., Cao Y., Liu H., Zhang Y., Wang J.G. (2022). Encapsulating ultrafine cobalt sulfides into multichannel carbon nanofibers for superior Li-ion energy storage. J Power Sources.

[19] Yoshio M., Tsumura T., Dimov N. (2005). Electrochemical behaviors of silicon based anode material. J. Power Sources.

[20] Jana M., Singh R.N. (2019). A facile route for processing of silicon-based anode with high capacity and performance. Materialia.

[21] Nulu A., Nulu V., Sohn K.Y. (2021). Unified NCNT@rGO bounded porous silicon composite as an anode material for Lithium-ion batteries. Korean J. Chem. Eng..

[22] Kim M.K., Shin W.H., Jeong H.M. (2019). Protective carbon-coated silicon nanoparticles with graphene buffer layers for high performance anodes in lithium-ion batteries. Appl. Surf. Sci..

[23] Xue L., Xu G., Li Y., Li S., Fu K., Shi Q., Zhang X. (2013). Carbon-coated Si nanoparticles dispersed in carbon nanotube networks as anode material for lithium-ion batteries. ACS Appl. Mater. Interfaces.

[24] Bai Y., Cao X., Tian Z., Yang S., Cao G. (2021). A high-performance silicon/carbon composite as anode material for lithium ion batteries. Nano Ex..

[25] Nulu A., Nulu V., Sohn K.Y. (2020). Si/SiOx nanoparticles embedded in a conductive and durable carbon nanoflake matrix as an efficient anode for lithium-ion batteries. ChemElectroChem.

[26] Rousselot S., Gauthier M., Mazouzi D., Lestriez B., Guyomard D., Roue L. (2012). Synthesis of boron-doped Si particles by ball milling and application in Li-ion batteries. J. Power Sources.

[27] Li P., Hwang J.H., Sun Y.K. (2019). Nano/micro-structured silicon-graphite composite anode for high-energy density li-ion battery. ACS Nano.

[28] Domi Y., Usui H., Shimizu M., Kakimoto Y., Sakaguchi H. (2016). Effect of phosphorus-doping on electrochemical performance of silicon negative electrodes in lithium-ion batteries. ACS Appl. Mater. Interfaces.

[29] Kong M., Noh J., Byun D., Lee J. (2009). Electrochemical characteristics of phosphorus doped silicon and graphite composite for the anode materials of lithium secondary batteries. J. Electroceramics.

[30] Ke C.Z., Liu F., Zheng Z.M., Zhang H.H., Cai M.T., Li M., Yan Q.Z., Chen H.X., Zhang Q.B. (2021). Boosting lithium storage performance of Si nanoparticles via thin carbon and nitrogen/phosphorus co-doped two-dimensional carbon sheet dual encapsulation. Rare Met..

[31] Nulu A., Nulu V., Sohn K.Y. (2021). Effect of cobalt doping on enhanced lithium storage performance of nanosilicon. ChemElectroChem.

[32] Mahmood N., Zhu J., Rehman S., Li Q., Hou Y. (2015). Control over large-volume changes of lithium battery anodes via active–inactive metal alloy embedded in porous carbon. Nano Energy.

[33] Fleischauer M.D., Topple J.M., Dahn J.R. (2005). Combinatorial investigations of Si-M  ( M   =  Cr  +  Ni , Fe , Mn )  thin film negative electrode materials, *Electrochem*. Solid-State Lett..

[34] Zhang F.M., Zeng Y., Gao J., Liu X.C., Wu X.S., Du Y.W. (2004). Ferromagnetism in Mn-doped silicon. J. Magn. Magn. Mater..

[35] Ma S.B., Sun Y.P., Zhao B.C., Tong P., Zhu X.B., Song W.H. (2006). Magnetic and electronic transport properties of Mn-doped silicon. Solid State Commun..

[36] Kim Y.J., Kim M.H., Yang J.H., Park J.W. (2006). Electrochemical properties of aluminum-doped silicon films as anode materials for lithium-ion batteries. J. Korean Phys. Soc..

[37] Huang X., Mao S., Chang J., Hallac P.B., Fell C.R., Luo Y., Metz B., Jiang J., Chen. J. (2015). Improving cyclic performance of Si anode for lithium-ion batteries by forming an intermetallic skin. RSC Adv..

[38] Lin L., Ma Y., Xie Q., Wang L., Zhang Q., Peng D.L. (2017). Copper-nanoparticle-induced porous Si/Cu composite films as an anode for lithium ion batteries. ACS Nano.

[39] Lee H.Y., Lee S.M. (2002). Graphite–FeSi alloy composites as anode materials for rechargeable lithium batteries. J. Power Sources.

[40] Wang Z., Zhang X., Liu X., Wang Y., Zhang Y., Li Y., Zhao W., Qin C., Mukanova A., Bakenovd Z. (2020). Bimodal nanoporous NiO@Ni–Si network prepared by dealloying method for stable Li-ion storage. J. Power Sources.

[41] Zeng J., Peng C.Q., Wang R.C., Liu Y.J., Wang X.F., Liu J. (2019). Preparation of dual-shell Si/TiO2/CFs composite and its lithium storage performance. Trans. Nonfeer. Met. Soc..

[42] Mulaudzi I., Zhang Y., Ndlovu G.F., Wu Y., Legodi M.A., Ree T. (2020). Copper doped Li_3_VO_4_ as anode material for lithium-ion batteries. Electroanalysis.

[43] Mueller F., Bresser D., Chakravadhanula V.S.K., Passerini S. (2015). Fe-doped SnO_2_ nanoparticles as new high capacity anode material for secondary lithium-ion batteries. J. Power Sources.

[44] Liu X., Li G., Zhang D., Chen D., Wang X., Li B., Li L. (2019). Fe-doped Li_3_VO_4_ as an excellent anode material for lithium-ion batteries: Optimizing rate capability and cycling stability. Electrochim. Acta.

[45] Zhang W., Zhou W., Wright J.H., Kim Y.N., Liu D., Xiao X. (2014). Mn-doped TiO_2_ nanosheet-based spheres as anode materials for lithium-ion batteries with high performance at elevated temperatures. ACS Appl. Mater. Interfaces.

[46] Tang X., Hou X., Yao L., Hu S., Liu X., Xiang L. (2014). Mn-doped ZnFe_2_O_4_ nanoparticles with enhanced performances as anode materials for lithium-ion batteries. Mater. Res. Bull..

[47] Nulu A., Nulu V., Sohn K.Y. (2022). Influence of transition metal doping on nano silicon anodes for Li-ion energy storage applications. J. Alloys Compd..

[48] Yi R., Dai F., Gordin M.L., Sohn H., Wang D. (2013). Influence of silicon nanoscale building blocks size and carbon coating on the performance of micro-sized Si–C composite Li-ion Anodes. Adv. Energy Mater..

[49] Shen D., Huang C., Gan L., Liu J., Gong Z., Long M. (2018). Rational design of Si@SiO_2_/C composites using sustainable cellulose as a carbon resource for anodes in lithium-ion batteries. ACS Appl. Mater. Interfaces.

[50] Xiang T., Chen Z., Rao Z., Yan M., Feng Z., Li X., Yang H., Huang J., Xing Shen X. (2021). Hierarchical Fe/Fe3C/C nanofibers as anodes for high capacity and rate in lithium ion batteries. Ionics.

[51] Chen J., Zhu B., Sun Y., Yin S., Zhu Z., Li J. (2018). Investigation of low-temperature selective catalytic reduction of NO_x_ with ammonia over Mn-modified Fe_2_O_3_/AC catalysts. J. Braz. Chem. Soc..

[52] Yang L.Y., Li H.Z., Liu J., Sun Z.Q., Tang S.S., Lei M. (2015). Dual yolk-shell structure of carbon and silica-coated silicon for high-performance lithium-ion batteries. Sci. Rep..

[53] Ding N., Xu J., Yao Y.X., Wegner G., Fang X., Chen C.H., Lieberwirth I. (2009). Determination of the diffusion coefficient of lithium ions in nano-Si. Solid State Ion..

[54] Reuter F., Baasner A., Pampel J., Piwko M., Dörfler S., Althues H., Kaskel S. (2019). Importance of capacity balancing on the electrochemical performance of Li[Ni_0.8_Co_0.1_Mn_0.1_]O_2_ (NCM811)/silicon full cells. J. Electrochem. Soc..

