# Supplementary material for: Metal (Cu/Fe/Mn)-Doped Silicon/Graphite Composite as a Cost-Effective Anode for Li-Ion Batteries
Source: Nanomaterials (Basel). 2022 Aug 30;12(17):3004. doi: 10.3390/nano12173004 (PMC9458242; doi:10.3390/nano12173004)
Supplement: Supplementary file 1 [file nanomaterials-12-03004-s001.zip › nanomaterials-1846405-supplementary.pdf]

Metal (Cu/Fe/Mn)-Doped Silicon/Graphite Composite as a Cost-Effective Anode for Li-Ion Batteries

Arunakumari Nulu, Young Geun Hwang, Venugopal Nulu and Keun Yong Sohn *

Department of Nanoscience and Engineering, Center for Nano Manufacturing, Inje University, 197 Inje-ro, Gimhae 50834, Gyeongnam-do, Korea

* Correspondence: ksohn@inje.ac.kr; Tel.: +82-55-320-3714

Supporting information

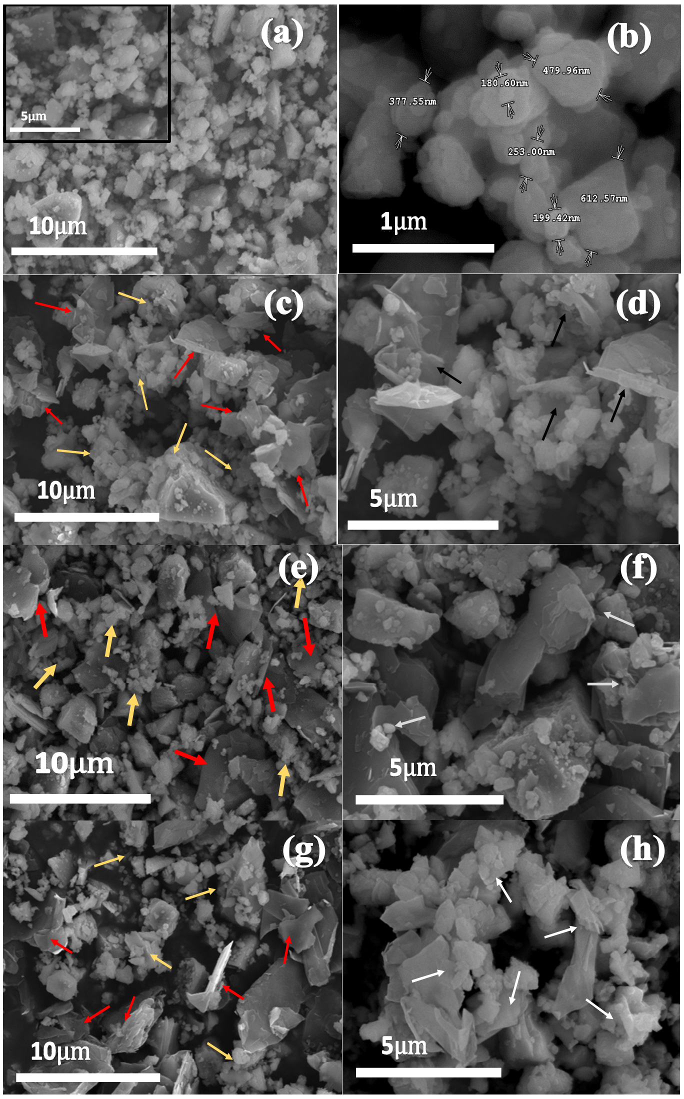

Figure S1. SEM images (a) & (b) Si- 48, (c) & (d) SiCuG, (e) & (f) SiFeG, and (g) & (h) SiMnG respectively.

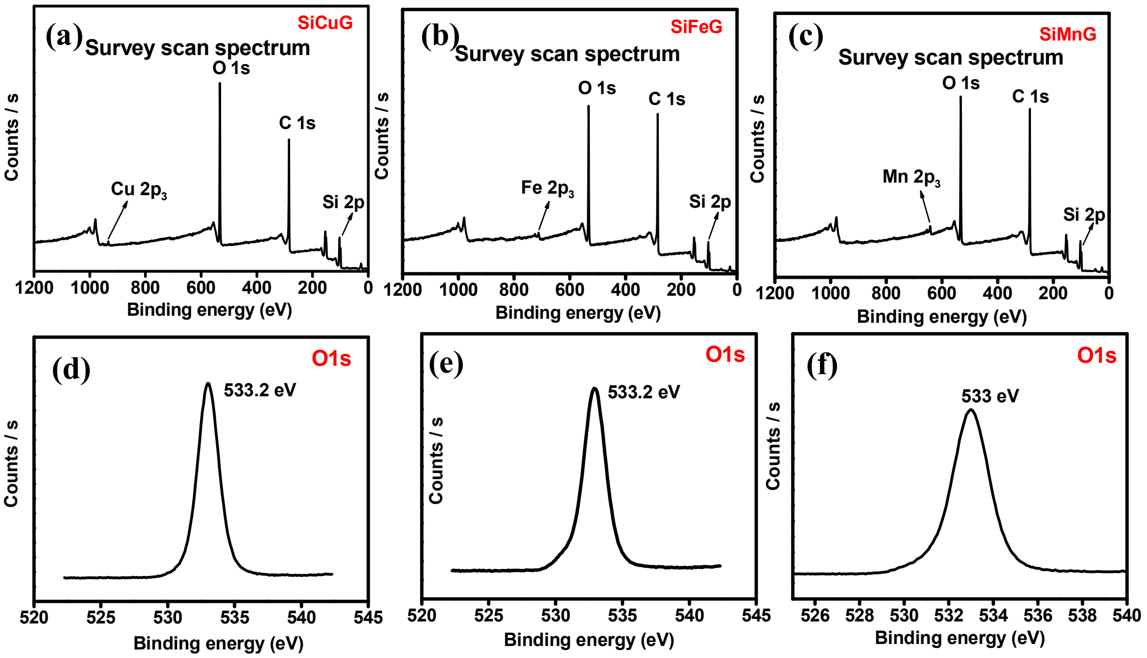

Figure S2. XPS-Spectra (a), (b), (c) survey scan spectra, (d), (e), (f) O 1S spectra of SiCuG, SiFeG, and SiMnG respectively.

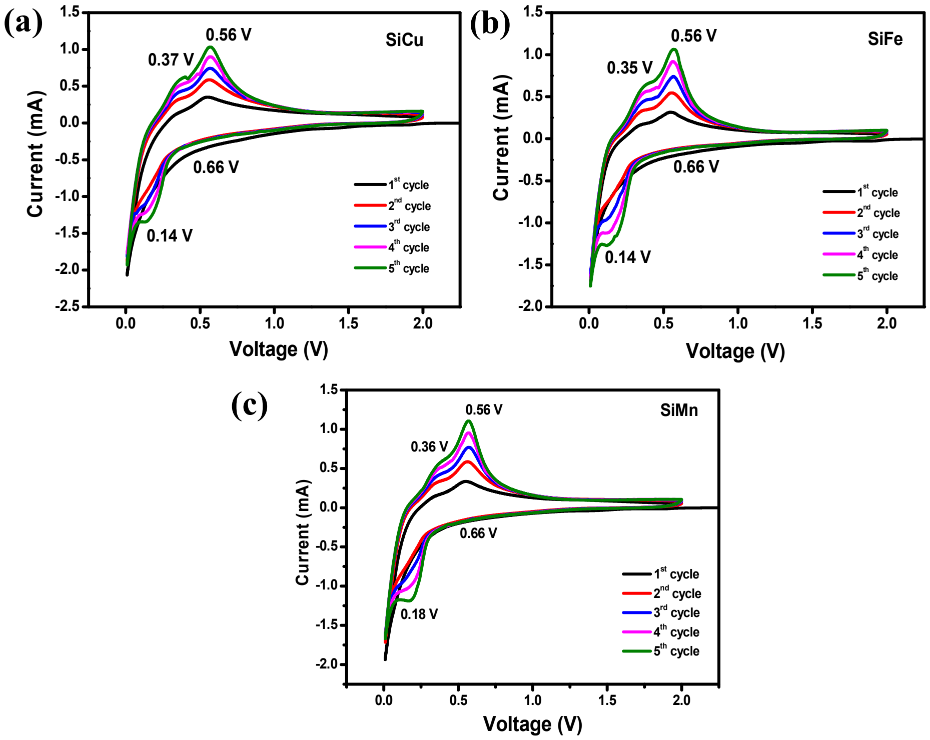

Figure S3. Cyclic voltammograms of (a) SiCu (b) SiFe and (c) SiMn vs. Li/Li^+ counter electrode with 1M $LiPF_6$ in EC: DEC: FEC (v/v ratio of 5:70:25) electrolyte at 0.1 mV s^{-1} scan rate, in the potential window of 0.01–2.0 V.

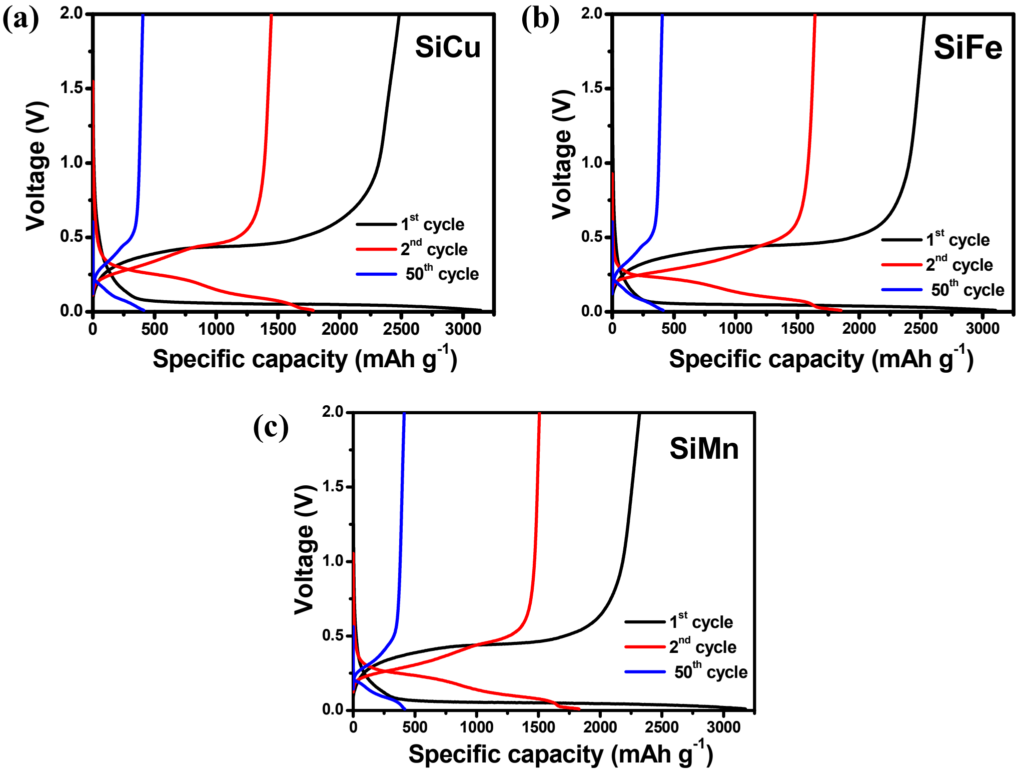

Figure S4. Voltage vs. specific capacity plots for 1st, 2nd, and 50th cycles (a) SiCu (b) SiFe and (c) SiMn vs. Li/Li^+ as a counter electrode with 1M LiPF6 in EC: DEC: FEC (v/v ratio of 5:70:25), at 200 mA g^{-1} in the potential window of 0.01–2.0 V.

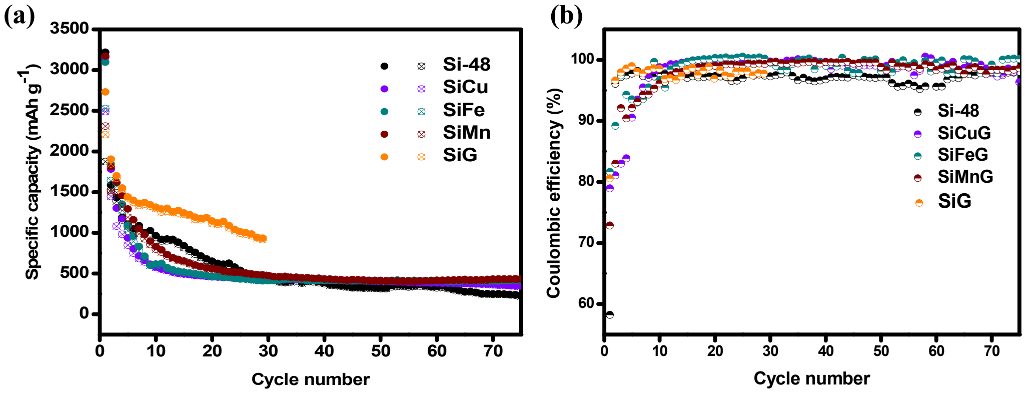

Figure S5. (a) cyclability (b) coulombic efficiency results of Si-48, SiCu, SiFe, SiMn and SiG vs. Li/Li^+ as counter electrode with 1M $LiPF_6$ in EC: DEC: FEC (v/v ratio of 5:70:25) electrolyte at 200 mA g^{-1} in the potential window of 0.01–2.0 V.

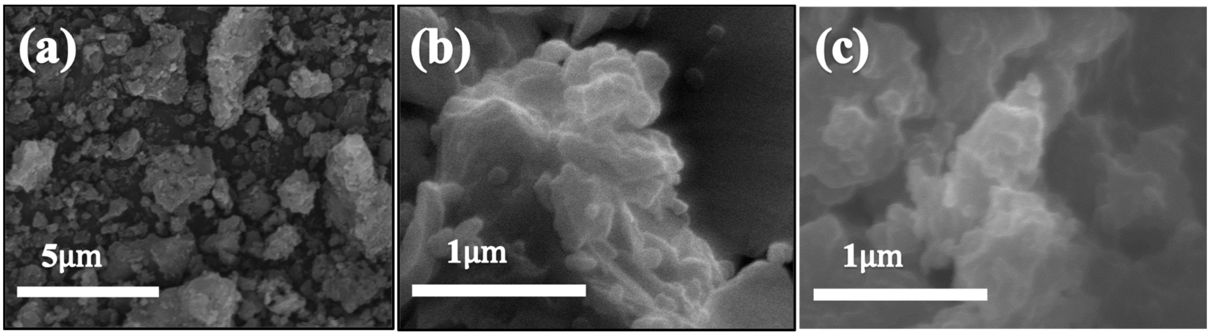

Figure S6. SEM images of Si-48 after 20 cycles at different magnifications

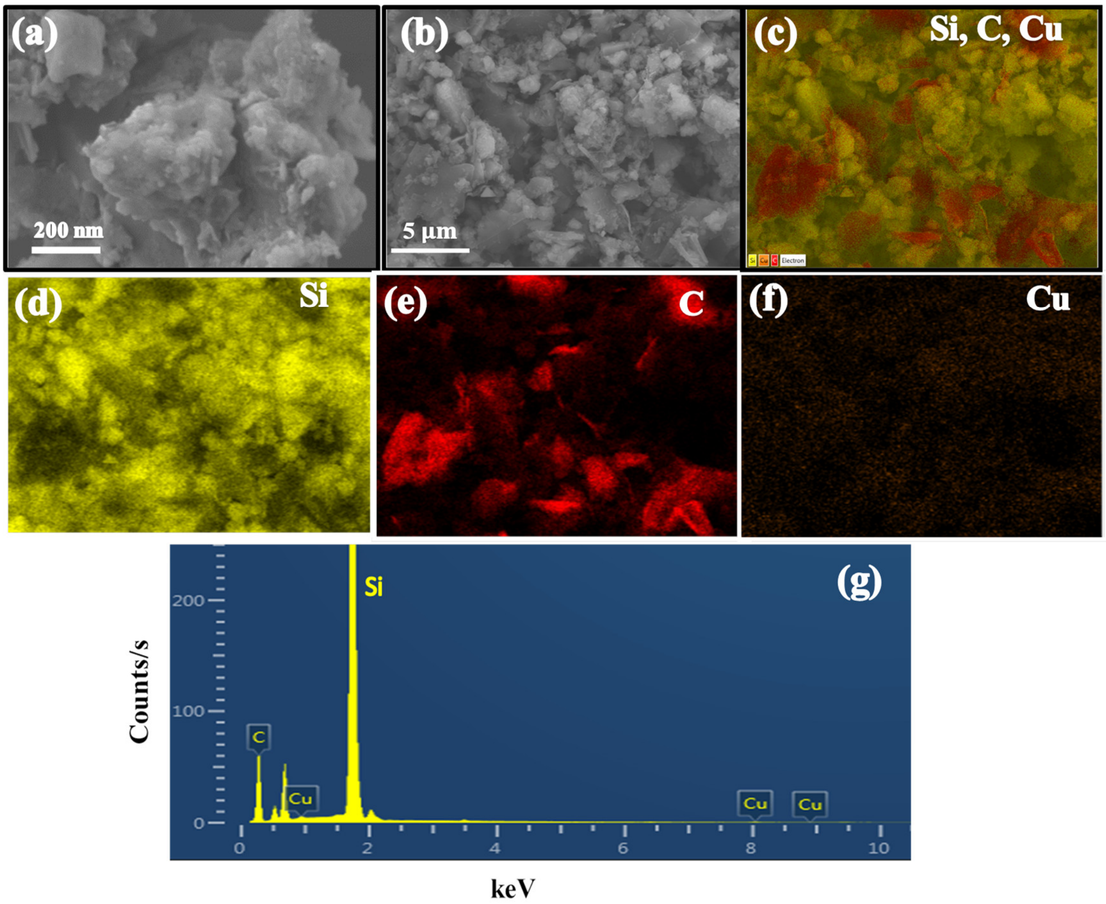

Figure S7. (a) and (b) SEM images (c) SEM-EDX elemental mapping of image (b) with respective Si, C, and Cu elements overlap (d) Si mapping (e) C mapping (f) Cu mapping (g) SEM-EDS spectra of image (b).

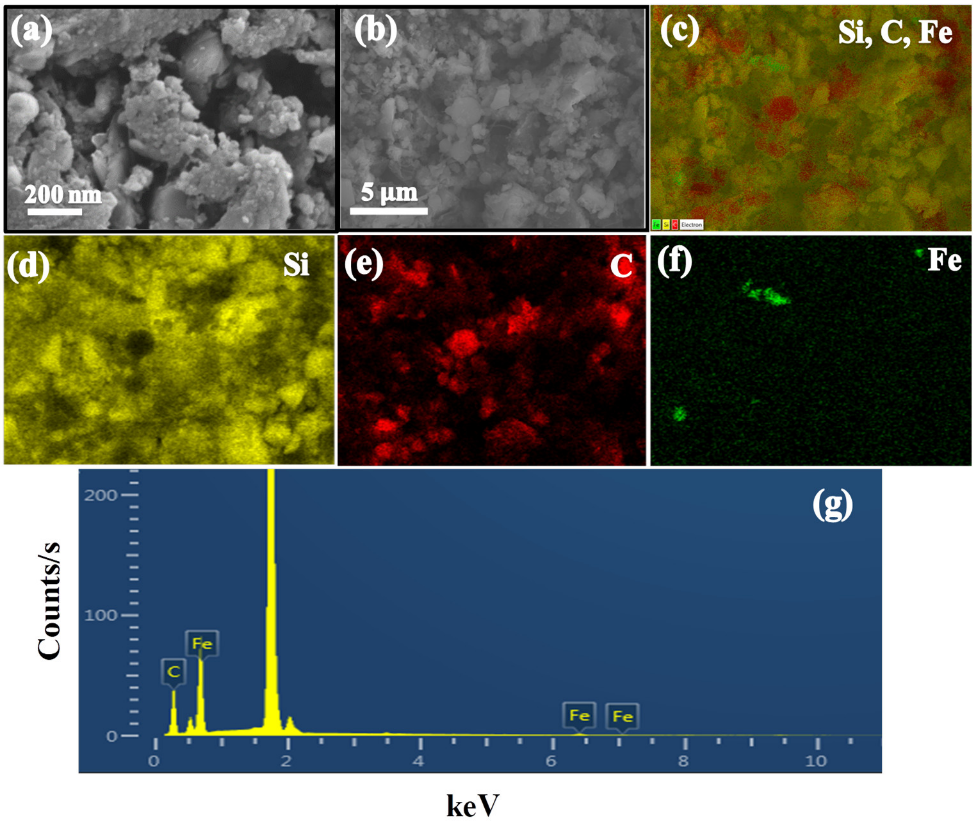

Figure S8. (a) and (b) SEM images (c) SEM-EDX elemental mapping of image (b) with respective Si, C, and Fe elements overlap (d) Si mapping (e) C mapping (f) Fe mapping (g) SEM-EDS spectra of image (b).

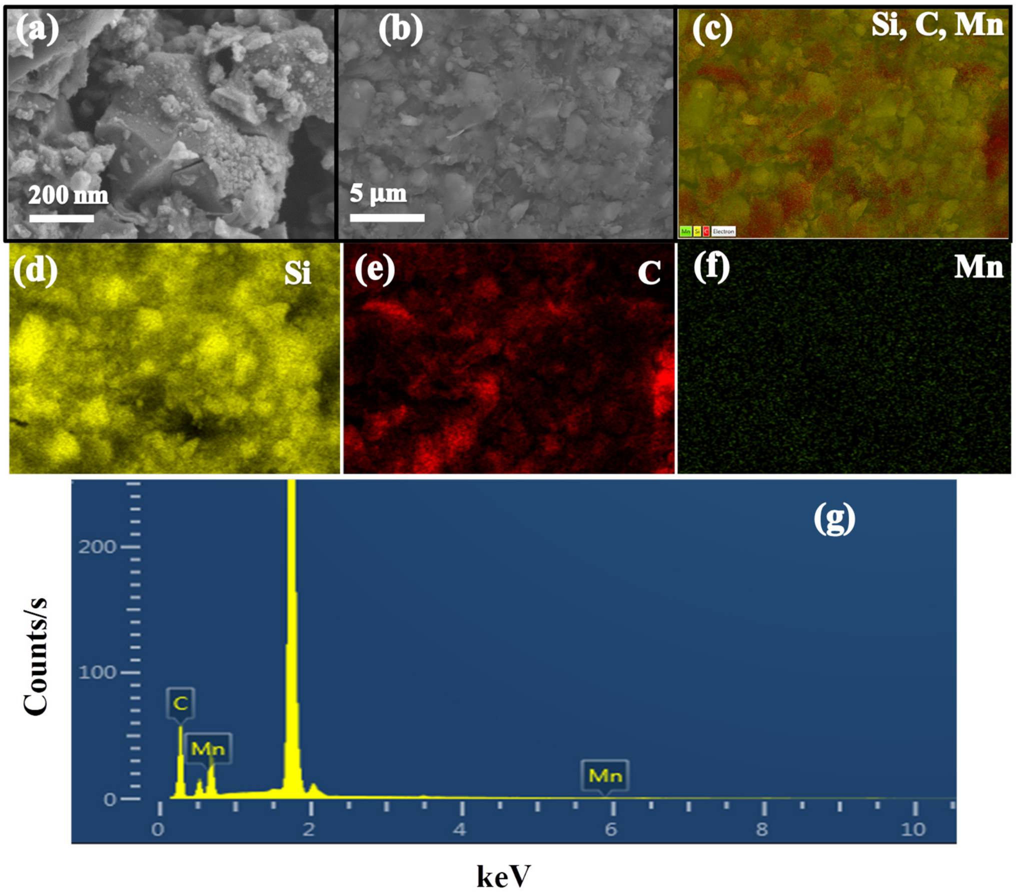

Figure S9. (a) and (b) SEM images (c) SEM-EDX elemental mapping of image (b) with respective Si, C, and Mn elements overlap (d) Si mapping (e) C mapping (f) Mn mapping (g) SEM-EDS spectra of image (b).

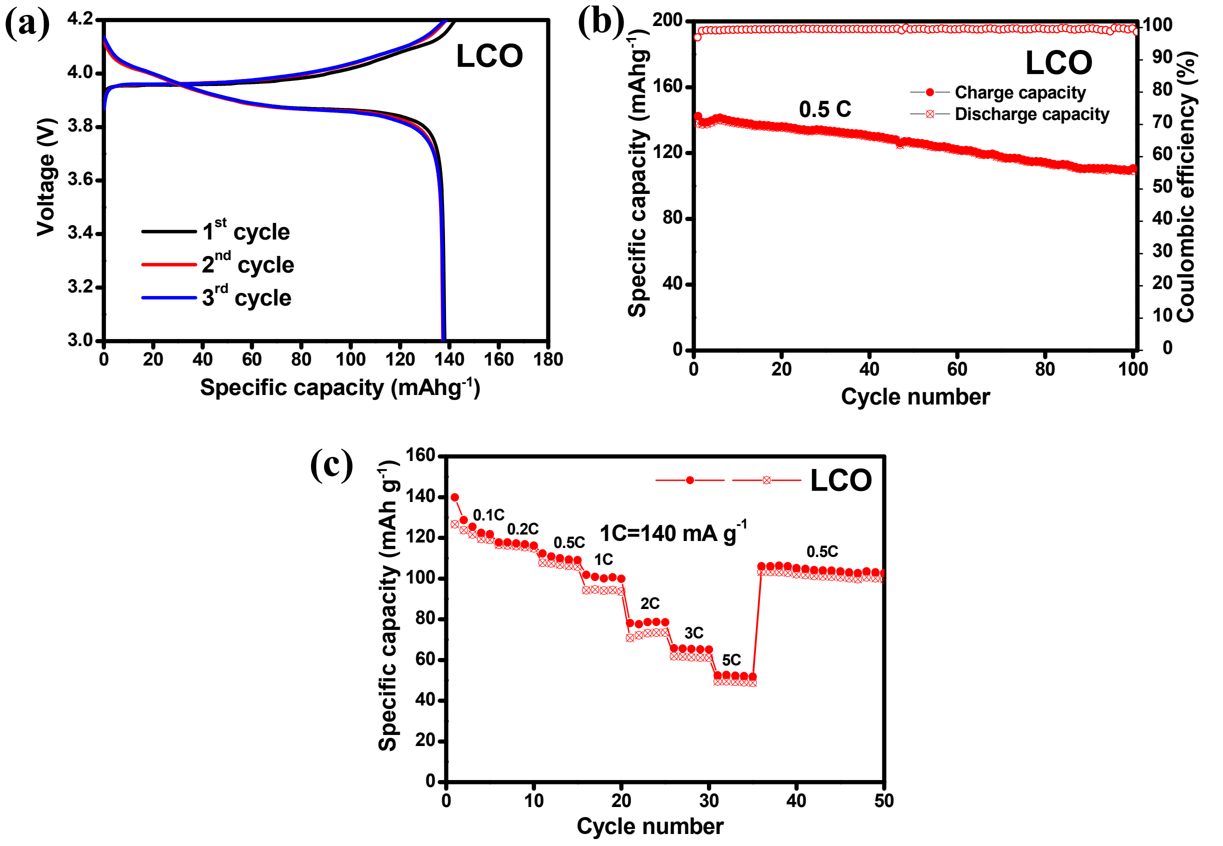

Figure S10. (a) Potential vs. specific capacity plots (b) cyclability (c) rate capability results of LCO vs. Li/L^+ as a counter electrode at 0.5 C, in the voltage window of 3.0–4.2 V.
